# *Akkermansia muciniphila*‐derived hypoacylated rough‐type lipopolysaccharides alleviate diet‐induced obesity via activation of TLR4−IL‐23−IL‐22 immune axis

**DOI:** 10.1002/imt2.70066

**Published:** 2025-07-17

**Authors:** Li Sun, Yuting Zhang, Wang Dong, Jingzu Sun, Tao Wang, Fei Shao, Huanqin Dai, Junjie Han, Wenzhao Wang, Shuo Wang, Tong Zhao, Liangliang Wang, Chang Liu, Shuangjiang Liu, Hongwei Liu

**Affiliations:** ^1^ State Key Laboratory of Microbial Diversity and Innovative Utilization Institute of Microbiology, Chinese Academy of Sciences Beijing People's Republic of China; ^2^ Medical School University of Chinese Academy of Sciences Beijing People's Republic of China; ^3^ CAS Key Laboratory of Pathogen Microbiology and Immunology, Institute of Microbiology Chinese Academy of Sciences Beijing People's Republic of China; ^4^ Institution Center for Shared Technologies and Facilities, Institute of Microbiology Chinese Academy of Sciences Beijing People's Republic of China; ^5^ The Laboratory of Microbiome and Microecological Technology, Institute of Microbiology Chinese Academy of Sciences Beijing People's Republic of China; ^6^ State Key Laboratory of Microbial Technology Shandong University Qingdao People's Republic of China

**Keywords:** *Akkermansia muciniphila*, antiobesity, hypoacylated rough‐type LPS, TLR4−IL‐23−IL‐22 immune axis

## Abstract

Lipopolysaccharides (LPS) derived from intestinal symbionts plays a critical role in modulating and maintaining mucosal immunity. In this study, we investigated the chemical characteristics and antiobesity properties of *Akkermansia muciniphila* HW07 LPS (ALPS). ALPS was identified as hypo‐acylated, *mono*/*bis*‐phosphorylated, rough‐type LPS. Compared to *Escherichia coli* LPS (ELPS), ALPS functions as a weak agonist of TLR4/TLR2. Intraperitoneal administration of ALPS in diet‐induced obese (DIO) mice suppressed weight gain, improved metabolic parameters, restored gut barrier integrity, and modulated the gut microbiota. Notably, ALPS treatment significantly increased plasma interleukin (IL)‐22 levels. Furthermore, neutralizing IL‐22 with an antibody eliminated the antiobesity effects of ALPS in DIO mice. Mechanistically, ALPS upregulated the expression of both IL‐22 and its upstream cytokine IL‐23 in a TLR4‐dependent manner. These findings confirm that activation of the TLR4−IL‐23−IL‐22 immune axis is a key mechanism underlying the antiobesity effect of ALPS. In acute toxicity assessment, no fatalities were observed in ALPS‐treated mice, whereas ELPS treatment led to a 40% mortality rate. Collectively, our results demonstrate that hypo‐acylated LPS from *A. muciniphila* functions as a metabolically beneficial immune modulator that exerts immunomodulatory effects through the TLR4−IL‐22 axis and suggests ALPS as a promising novel therapeutic strategy for metabolic disorders.

## Highlights

- *Akkermansia muciniphila* produces the rough‐type lipopolysaccharides composed of the tetra‐acylated and *mono/bis*‐phosphorylated lipid A species.
- ALPS administration alleviates obesity and metabolic disorders, restores gut barrier function, and reshapes gut microbiota in DIO mice.
- The metabolic benefits of ALPS are mechanistically linked to the activation of the TLR4−IL‐23−IL‐22 immune axis.

## Graphical abstract

This study demonstrates that hypoacylated, rough‐type lipopolysaccharides (LPS) derived from *Akkermansia muciniphila* strain HW07 (ALPS) alleviates diet‐induced obesity. ALPS acts as a weak TLR4 agonist and significantly reduces weight gain, improves metabolic parameters, and restores gut barrier integrity in obese mice. The antiobesity effects are mediated primarily through activation of the TLR4−IL‐23−IL‐22 immune axis, leading to increased IL‐22 production. ALPS also reshapes the gut microbiota (e.g., increases SFB, *C. cocleatum*) and elevates beneficial short‐chain fatty acids. These findings identify *A. muciniphila* LPS as a safe and effective therapeutic candidate for metabolic disorders via TLR4‐dependent IL‐22 induction. The diagram was created using Microsoft PowerPoint and https://BioRender.com.

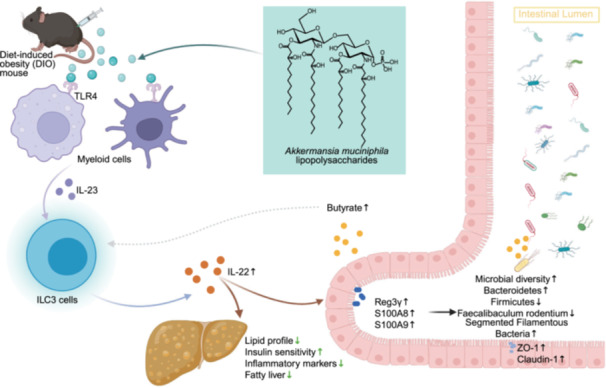

## INTRODUCTION

In recent decades, experimental and clinical studies have definitively confirmed the causal relationship between gut microbiota dysbiosis and metabolic diseases [1, 2, 3]. Evidence demonstrates that supplementation with gut commensals, which typically decrease in abundance during obesity, diabetes, and cardiovascular diseases, aids in preventing and alleviating these metabolic conditions [4, 5, 6]. Among these gut bacteria, *Akkermansia muciniphila* stands out due to its high abundance in healthy individuals and its potent therapeutic effects on metabolic diseases. Early cohort studies have shown negative correlations between the abundance of *A. muciniphila* and conditions such as obesity, diabetes, atherosclerosis, and colitis [7, 8]. The administration of live or pasteurized *A. muciniphila* in animal studies effectively reduces obesity and atherosclerosis, improves insulin resistance, and enhances anti‐PD‐1 immunotherapy [9, 10, 11, 12, 13, 14]. Importantly, its benefits for overweight and obese individuals have been validated in a clinical trial [15]. Several bioactive components of *A. muciniphila* contributing to its probiotic efficacy have been identified, including the outer membrane protein Amuc_1100, the secreted protein Amuc_1409, branched diacyl phosphatidylethanolamine, tripeptide, and threonyl‐tRNA synthetase [9, 16, 17, 18, 19]. Products derived from *A. muciniphila* claiming health benefits are already marketed in Australia.

Lipopolysaccharides (LPS) is an essential component of Gram‐negative bacterial outer membranes. LPS not only mediates direct interactions between gut bacteria and intestinal immune cells but also influences systemic immune responses by entering the circulation. LPS shares a conserved structural “pattern,” composed of lipid A and a heteropolysaccharide domain. The heteropolysaccharide domain is divided into a membrane‐proximal core oligosaccharide (core OS) and a repeating O‐polysaccharide domain (O‐chain). The chemical structure of LPS significantly influences their effects on immune response. Recent studies highlighted variations in the chemical structures of gut microbiota‐derived LPS and their differing physiological effects. For example, LPS from *Bacteroides dorei* enhances immunological tolerance [20], LPS from *Bacteroides vulgatus* reduces response to subsequent LPS‐stimuli and alleviates intestinal inflammation in mice [21, 22], LPS from *Rhodobacter sphaeroides* improves insulin sensitivity [23], and LPS from *Parabacteroides goldsteinii* alleviates chronic obstructive pulmonary disease [24].

Cytokines secreted by immune cells serve important function in responding to pathogens and external stimuli, maintaining barrier integrity, and repairing tissue injuries. Among these, IL‐22, a member of the IL‐10 cytokine family, plays a pivotal role in orchestrating comprehensive mucosal defense by stimulating the secretion of antimicrobial peptides, enhancing mucus production, and promoting the expression of tight junction proteins [25]. IL‐22 contributes to gut microbiota homeostasis by fostering the colonization of symbiotic bacteria while inhibiting the overgrowth of pathobiont bacteria [26]. In the intestine, IL‐22 is primarily produced by RORγt‐expressing group 3 innate lymphoid cells (ILC3s) [27]. However, long‐term high‐fat diet (HFD) exposure impairs the functionality of intestinal ILC3s, leading to diminished IL‐22 production [28]. This deficiency creates a permissive microenvironment that promotes epithelial damage and gut microbiota dysbiosis, potentially contributing to the increased intestinal permeability observed in metabolic syndrome. Exogenous administration of IL‐22 produces multiple metabolic benefits in leptin receptor‐deficient (*db/db*) mice, including the enhanced insulin sensitivity, restored gut barrier function, and reduced enteric inflammation [29].

**Figure 1:**
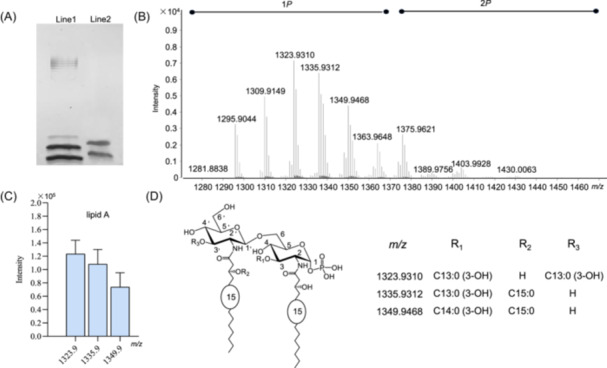
The silver‐stained sodium dodecyl sulfate polyacrylamide gel electrophoresis (SDS‐PAGE) and the representative chemical structure of lipid A. (A) Silver staining of SDS‐PAGE with lipopolysaccharide (LPS). Lanes 1 and 2 are LPS from *Escherichia coli* O111:B4 (1 mg/mL, 8 μL,) and *Akkermansia muciniphila* HW07 (5 mg/mL, 4 μL), respectively. (B) Negative‐ion mode ESI‐MS spectrum of the lipid A from *A. muciniphila* HW07. (C) Relative quantitation of the lipid A species at *m/z* 1323.9, 1335.9, and 1349.9 from *A. muciniphila* HW07 using LC‐MS. Ion intensities were extracted at the monoisotopic masses. (D) Structure of the lipid A with molecular weight of 1323.9, 1335.9, and 1349.9. The lipid A is heterogeneous, composed of a mixture of tetra‐acylated *mono‐* or *bis‐*phosphorylated species (P, phosphate group). 1P: *mono*‐phosphorylated lipid A species. 2P: *bis*‐phosphorylated lipid A species.

Pasteurized *Akkermansia muciniphila* have demonstrated notable efficacy in improving obesity and diabetes. However, whether LPS contribute to the therapeutic effects of inactivated *A. muciniphila* remains uncertain. In this study, we first investigated the chemical properties of LPS derived from *A. muciniphila* HW07 (termed as ALPS), which was isolated from a healthy human donor in China and cultured in a mucin‐based medium. ALPS was chemically defined as a tetra‐acylated rough type LPS (Figure 1) and further identified as a weak TLR4/TLR2 agonist, inducing moderate immunomodulatory response. Administration of ALPS in HFD‐induced obese mice reduced body weight gain, alleviated metabolic disturbances, restored gut barrier integrity, and reshaped the gut microbiota composition. Regarding cytokine profiles, ALPS treatment significantly increased IL‐22 and IL‐23 secretion. Further investigation using anti‐IL‐22 antibody in high fat diet‐fed mice and bioassay in *Tlr4* ^*−*/*−*^ mice demonstrate that activation of TLR4–IL‐23–IL‐22 axis as a key underlying mechanism for the antiobesity effects of ALPS.

## RESULTS

### Purification and characterization of *A. muciniphila* LPS

In this study, we extracted *A. muciniphila* HW07 LPS using an established method [30], with rigorous removal of cellular contaminants, including proteins, nucleic acids, lipoproteins, and phospholipids. SDS‐PAGE analysis confirmed that ALPS is a rough‐type LPS (R‐LPS) (Figure 1A). The predominant fatty acid detected was 3‐hydroxypentadecanoic acid (C15:0 (3‐OH)) (Figure S1). The l‐fucose, 2‐deoxy‐2‐amino‐d‐galactose, 2‐deoxy‐2‐amino‐d‐glucose, d‐glucose, d‐mannose, heptose, and 3‐deoxy‐d‐manno‐2‐oct‐ulopyranosonic acid were found in the ALPS by acid hydrolysis and monosugar analysis (Figure S2) [31].

**Figure 2:**
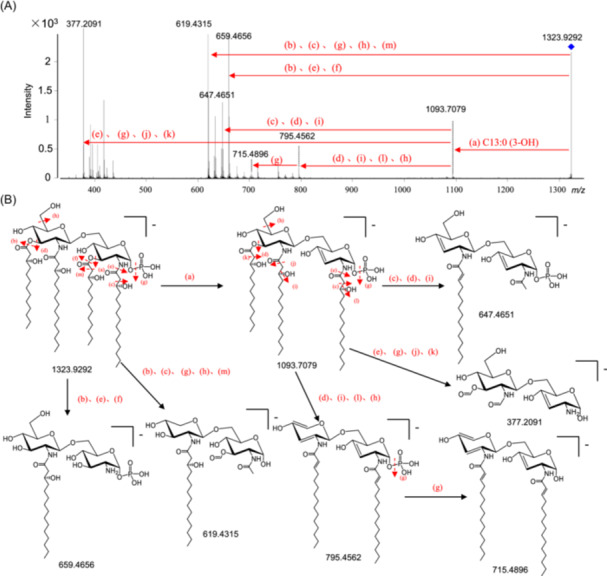
HPLC‐ESI‐MS^2^ spectroscopic analysis of the lipid A in *Akkermansia muciniphila* LPS (ALPS). (A) Negative‐ion mode MS/MS spectrum of the ion at *m/z* 1323.9292. (B) Proposed fragmentation scheme of the ion at *m/z* 1323.9292.

To attain structural information, the lipid A moieties and de‐acylated oligosaccharides were prepared from ALPS. The main lipid A species were purified and characterized by HPLC‐ESI‐MS^2^ data analysis (Figure S3). In detail, a cluster of peaks in the mass range of 1281–1430 Da was observed (Figure 1B), indicating a high heterogeneity of lipid A. The relative abundance of lipid A species corresponding to 1323.9, 1335.9, and 1349.9 based on LC‐MS data was presented (Figure 1C). Of the most intense ion peak at *m/z* 1323.93, fragment ions at *m/z* of 377.20, 619.43, 647.46, 659.46, 715.48, 795.45, and 1093.70 confirmed the presence of a *mono*‐phosphorylated and tetra‐acylated lipid A, with two C15:0 (3‐OH) fatty acid chains as N‐linked acyl chains and two C13:0 (3‐OH) fatty acid chains as primary O‐linked fatty acids by referring to the fragmentation rules and the biosynthesis of lipid A (Figures 1D and 2). The fragment ions at *m/z* 619.43 or 659.46 are diagnostic of C15:0 (3‐OH) for the 2/2′‐N‐linked acyl chains. By the similar way, the structures of lipids A corresponding to 1335 and 1349 were also determined (Figures 1D, S4, and S5). They possess a secondary fatty acid on the 2′‐N‐linked chain, while lacking the 3′‐O‐linked acyl chain. In addition, the lipid A species at *m/z* 1295.90, 1309.91, and 1363.96 were indicated to be *mono*‐phosphorylated lipid A (Figure S6A–C), and the lipid A species at *m/z* 1375.96, 1389.97, 1403.99, and 1430.00 were determined to be *bis*‐phosphorylated lipid A in consideration of their precursor ions and the key fragment ions (Figure S6D–G). Thus, LPS in the *A. muciniphila* HW07 strain cultured in a mucin‐based medium are mainly composed with the *mono*‐phosphorylated and tetra‐acylated lipid A (Figure 1). In the following, we sequenced the genome of strain HW07 and further compared the biosynthetic gene cluster of lipid A between *E. coli* MG1655 and the strain HW07 (Table S1). Of the lipid A gene cluster in HW07, homologue genes of *lpxA, lpxB, lpxC, lpxD*, and *lpsK* with a relatively high identity are predicted on the basis of amino acid sequence similarity, supporting the synthesis of 2/2′, 3/3′ tetra‐acylated lipid A skeleton. In addition, the gene *JFJHEM_01585* is deduced to be a potentially new acyltransferase for the secondary fatty acid on 2′‐acyl chain due to its low identity with *lpxL* (23.7%) or *lpxP* (21.9%). However, the *lpxM* homologue gene that is responsible for transfer of the secondary fatty acid chain on the 3′‐acyl chain is absent. Above genome analysis evidence the production of hypo‐acylated lipid A in the LPS of *A. muciniphila*.

The de‐acylated oligosaccharides were subjected to in‐source fragmentation‐based HPLC‐ESI‐MS analysis. The double‐charged ions at *m/z* 869.76, 909.74, 950.29, 990.28, 1184.90, 1224.88, and 1622.51 showed seven distinct of oligosaccharides, OS1‐OS7 (Figure S7). The fragment ions (*m/z* 1466.37, 1320.42, 1100.35, 939.20, 719.14, and 499.07) from the *mono‐*charged molecular ion at *m/z* 1820.52 in the ESI‐MS of OS2 by in‐source fragmentation, along with the fragment ions (*m/z* 828.72, 732.68, 622.65, 469.09, and 359.06) from the double‐charged molecular ion at *m/z* 909.74, showed the presence of three 3‐deoxy‐D‐*manno*‐2‐oct‐ulopyranosonic acid moieties (Kdo), three hexosamines, one fucose, one heptose, one hexose, two phosphoric acid moieties (Figure S8A). According to the reported MS fragmentation pattern of LPS, oligosaccharides OS2, OS4, OS6, and OS7 were indicated to have the *bis*‐phosphorylated disaccharide on the basis of fragment ions at *m/z* 499, 719, and 939 ions in the ESI‐MS spectrum (Figure S8A,D,F,G); oligosaccharides OS1, OS3, OS5 were deduced to have the *mono*‐phosphorylated disaccharide on the basis of their precursor ions and the characteristic fragment ions at *m/z* 419 and 859 (Figure S8B,C,E). Further chromatography separation on the de‐acylated oligosaccharides of LPS yielded one hexadecasaccharide OS7 whose MS and NMR data (Figures S8G, S9, and S10) matched with the reported oligosaccharide (OS_1deAc_) of the LPS from a European strain of *A. muciniphila* (Euro‐ALPS, ATCC BAA‐835) [31]. The structures for OS1‐OS6 remained unsolved due to challenges for purification.

### ALPS elicit a moderate immune response rather than an inflammatory response

Hypo‐acylated gut bacterial LPS have been shown to induce beneficial immunomodulatory responses in inflammatory bowel disease (IBD), chronic obstructive pulmonary disease (COPD), and obesity [21, 22, 23, 24]. In this study, we evaluated the immunological activities of ALPS using RAW 264.7 cells, bone marrow–derived dendritic cells (BMDCs), and bone marrow–derived macrophages (BMDMs). Compared to LPS from *E. coli* (ELPS), ALPS elicited significantly reduced levels of TNF‐α, IL‐6, and IL‐1β messenger RNA (mRNA) expression and comparable levels of IL‐10 in both BMDMs and BMDCs (Figure S11A–J). Interestingly, lipid A and the heteropolysaccharide domain alone failed to induce an immune response in RAW 264.7 cells. The in vitro results demonstrates that ALPS exhibits an immune‐regulatory effect rather than a proinflammatory response.

### ALPS protect mice from diet‐induced obesity and related metabolic disorders

**Figure 3:**
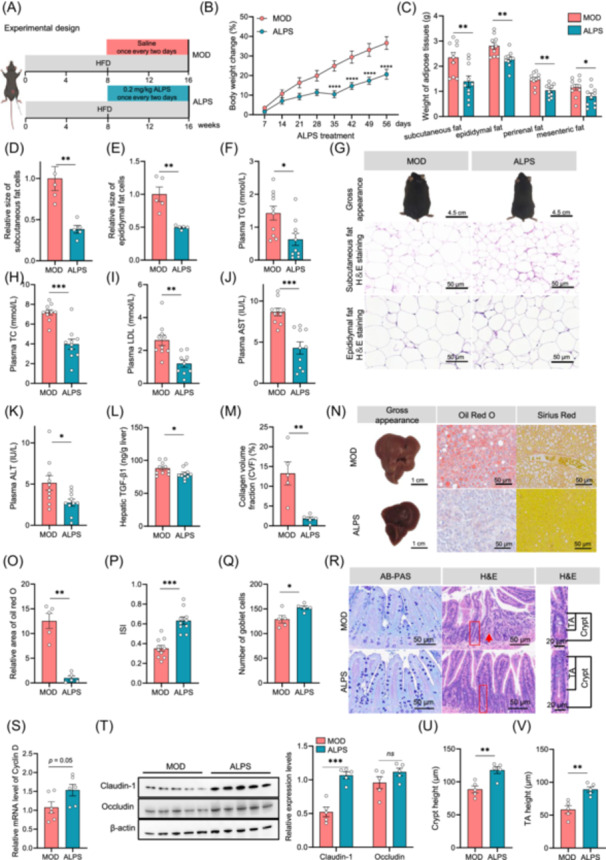
*Akkermansia muciniphila* LPS (ALPS) protects against metabolic disorders in diet‐induced obese (DIO) mice. (A) Experimental design showing groups and durations. MOD: mice fed a high‐fat diet (HFD), intraperitoneally injected with saline once every 2 days (0–16 weeks). ALPS: mice fed a high‐fat diet (HFD), intraperitoneally injected with 0.2 mg/kg ALPS once every 2 days (0–16 weeks). (B) Body weight changes over time. (C) Weight of adipose tissues. (D) Relative size of subcutaneous fat cells. (E) Relative size of epididymal fat cells. (F) Plasma triglyceride (TG). (G) Representative images showing the gross appearance of mice histology (scale bar, 4.5 cm) and hematoxylin and eosin (H&E)‐stain sections of adipose tissues (scale bar, 50 μm). (H) Plasma total cholesterol (TC). (I) Plasma low‐density lipoprotein (LDL). (J) Plasma aspartate aminotransferase (AST). (K) Plasma alanine aminotransferase (ALT). (L) Hepatic TGF‐β1 levels. (M) Collagen volume fraction (VCF). (N) Representative images of liver histology (scale bar, 1.0 cm) and Oil Red O and Sirius Red staining of liver tissue (scale bar, 50 μm). (O) Relative area of Oil Red O. (P) Insulin sensitivity index (ISI). (Q) Number of goblet cells. (R) Representative AB‐PAS (scale bar, 50 μm) and H&E‐stained images of pathological ileum sections (scale bar, 20 μm). (S) Relative messenger RNA expression of Cyclin D in the ileum. (T) Relative protein expression of Occludin and Claudin‐1 in the ileum. (U) Crypt height. (V) Transit‐amplifying (TA) zone height. MOD: Model group, high‐fat diet (HFD). ALPS: *A. muciniphila* lipopolysaccharides, (ALPS)‐treated HFD group. ^ns^ *p* > 0.05, *p < 0.05, **p < 0.01, ***p < 0.001. Welch's *t*‐test in D–E, K, M, and O. Unpaired *t‐*test in C, F, H–J, L, P and Q, and S–V. Two‐way ANOVA (Sidak) in (B). *n* values are as follows: (B, C, F, H, J–L, P) *n* = 10, (I) *n* = 9–10, (D, E, G, M–O, Q, R, T, U, V) *n* = 5, (S) *n* = 6.

To investigate whether ALPS mediates the antiobesity effect of *A. muciniphila*, diet‐induced obese mice (DIO) were intraperitoneally administered ALPS at a dose of 0.2 mg/kg once every 2 days (Figure 3A). This dosage, with slight modification, was based on a previous report [23]. ALPS treatment significantly mitigated HFD‐induced weight gain and associated metabolic disorders (Figures 3B and S12A). After 56 days, fat masses of subcutaneous, epididymal, perirenal, and mesenteric adipose tissues were reduced by 40.7%, 18.9%, 29.8%, and 31.0%, respectively (Figure 3C). These changes were accompanied by a decrease in adipocyte size in both subcutaneous and epididymal adipose tissue (Figure 3D–E,G). Plasma levels of triglycerides (TG), total cholesterol (TC), free fatty acids (FFA), and low‐density lipoprotein cholesterol (LDL‐C) were also significantly decreased by 55.9%, 44.8%, 15.6%, and 54.4%, respectively (Figures 3F,H–I, and S12B). Additionally, ALPS treatment alleviated obesity‐related liver dysfunction, as evidenced by reduced hepatic lipid levels, plasma aspartate aminotransferase (AST), alanine aminotransferase (ALT), and TGF‐β1, as well as improvements in liver index and hepatic collagen volume fraction (VCF) (Figures 3J–M and S12C–F). Oil red O staining further confirmed the beneficial effects of ALPS‐treated DIO mice (Figure 3N–O). Liver transcriptome analysis revealed the upregulation of 126 genes and downregulation of 70 genes including 24 involved in lipid metabolism (Figure S13A,B). Gene Ontology (GO) enrichment analysis indicated that downregulated genes in the ALPS‐treated group were significantly enriched in metabolic processes, such as monocarboxylic acid metabolic process, fatty acid metabolic process, and lipid metabolic process (Figure S13C). quantitative polymerase chain reaction (qPCR) analysis confirmed reduced expression of key genes involved in lipid metabolism dysfunction in the liver, including *Acot1, Acot3, Acot4, Cd36, Acacb, Hmgcs1, Cidea, Elovl6, Acox1, Lipe*, and *Pnpla2* (Figure S13D) [32, 33, 34, 35]. In contrast to HFD‐induced insulin resistance and glucose metabolism impairments, ALPS significantly improved oral glucose tolerance (OGTT), insulin tolerance (ITT), and insulin sensitivity index (ISI) (Figures 3P and S12G–M). Moreover, ALPS treatment significantly reduced plasma levels of LPS, TNF‐α, IL‐6, IL‐1β, MCP‐1, and the TNF‐α/IL‐10 ratio, while enhancing IL‐10 levels (Figure S12N–T).

Long‐term HFD feeding impairs the intestinal epithelial barrier, leading to sustained low‐grade inflammation during obesity development. ALPS treatment significantly increased goblet cell numbers, promoted intestinal epithelial cell proliferation and regeneration, and enhanced gut barrier function (Figure 3Q–V). Specifically, the mRNA level of cyclin D, a key regulator of intestinal cell proliferation, was upregulated 1.5‐fold compared to the model group (Figure 3S). Additionally, the expression of tight junction proteins Claudin‐1 and Occludin was significantly increased by ALPS (Figures 3T and S12U). ALPS treatment also restored the heights of crypt and transit‐amplifying (TA) (Figure 3U,V). Collectively, these findings support the conclusion that ALPS treatment enhances gut barrier integrity.

### Increased IL‐22 contributes to the antiobesity effect of ALPS

**Figure 4:**
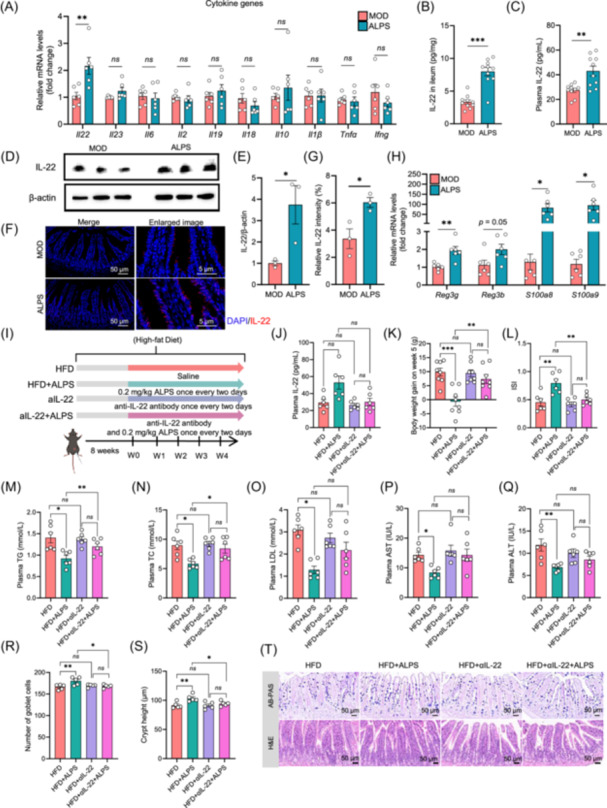
*Akkermansia muciniphila* LPS (ALPS) promotes IL‐22 production in diet‐induced obese mice. (A) The expression of cytokine genes in ileum. (B) IL‐22 levels in the ileum. (C) IL‐22 levels in plasma. (D) Western blot analysis of IL‐22 and β‐actin expression in the ileum. (E) Relative expression levels of IL‐22 in intestinal cells. (F) Immunofluorescent staining of IL‐22 in the ileum (scale bar, 50 μm in main panel; 5 μm in enlarged image). (G) The mean fluorescence intensity of IL‐22. (H) Expression of antimicrobial peptide genes (fold change). (I) Experimental design showing groups and durations. aIL‐22, high‐fat fed mice treated with anti‐IL‐22. aIL‐22+ALPS, high‐fat fed mice treated with anti‐IL‐22; HFD, high‐fat fed mice. HFD+ALPS, high‐fat fed mice treated with ALPS. (J) Plasma IL‐22 levels. (K) Body weight gain in week 5. (L) Plasma insulin sensitivity index (ISI). (M) Plasma triglyceride (TG). (N) Plasma total cholesterol (TC). (O) Plasma low‐density lipoprotein (LDL). (P) Plasma aspartate aminotransferase (AST). (Q) Plasma alanine aminotransferase (ALT). (R) Number of the goblet cells in the ileum. (S) Crypt height in the ileum. (T) Representative AB‐PAS and H&E staining images of pathological ileum sections (scale bar, 50 μm). MOD: Model group, high‐fat diet (HFD). ALPS: *A. muciniphila* lipopolysaccharides, (ALPS)‐treated HFD group. ^ns^ *p* > 0.05, *p < 0.05, **p < 0.01, ***p < 0.001. Welch's *t*‐test in A, B, and C. Unpaired *t‐*test in F–H, J–S. *n* values: (A, F, H, J, K–O) *n* = 6, (B, C) *n* = 10, (I) *n* = 8, (P–R) *n* = 5, (D, E) *n* = 3.

Given the critical role of gut mucosal immunity in defending against pathogenic bacteria and maintaining intestinal barrier integrity [36, 37], we investigated the effect of ALPS on cytokine production in intestinal epithelial cells using qPCR (Figure 4A). ALPS treatment led to a 2.2‐fold upregulation of *Il22* mRNA expression. Correspondingly, significantly elevated IL‐22 protein levels were detected in both the ileum and plasma (Figure 4B–G). IL‐22 is known to induce the expression of antimicrobial peptides in gut epithelial cells. In line with this, ALPS treatment upregulated the expression of antimicrobial peptide genes, including *Reg3g*, *S100a8*, and *S100a9* (Figure 4H). Notably, IL‐22 deficiency has been reported in obese mice [29], and exogenous IL‐22 supplementation has been shown to ameliorate hyperglycemia and insulin resistance [29, 38, 39]. To further determine the causal role of IL‐22 in the ALPS‐mediated metabolic benefits, we administrated ALPS‐treated and untreated DIO mice with the anti‐IL‐22 antibody (Figure 4I). Five‐week treatment with the antibody alone did not affect the plasma IL‐22 levels in antibody‐treated DIO mice, but resulted in a dramatic decrease in IL‐22 levels in mice treated with both ALPS and the antibody (Figure 4J). Importantly, the metabolic improvements and protection of gut barrier function induced by ALPS were largely abrogated by IL‐22 neutralization (Figures 4K–T and S14). These findings indicate that IL‐22 plays a central role in mediating the antiobesity and gut‐protective effects of ALPS.

### ALPS activates the TLR4‐IL‐23‐IL22 signaling pathway

**Figure 5:**
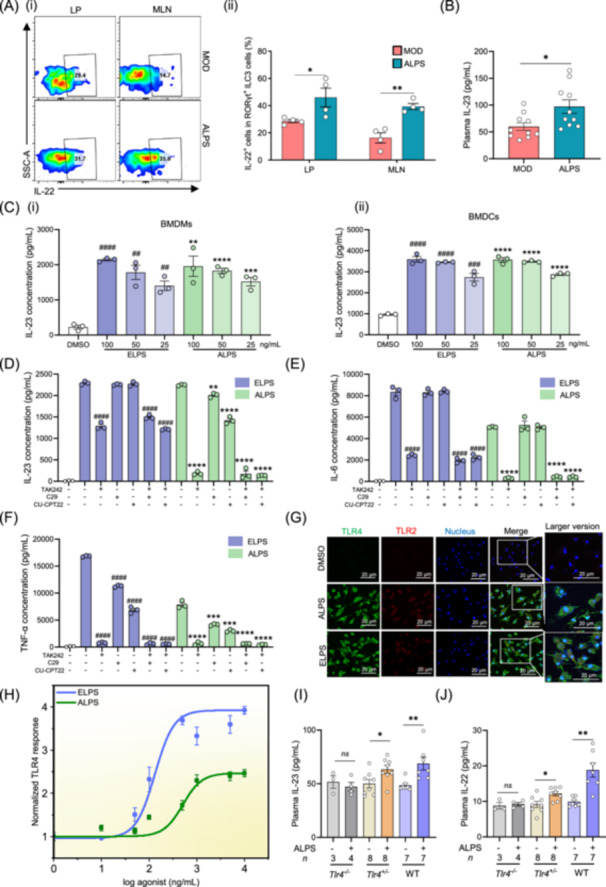
*Akkermansia muciniphila* LPS (ALPS) activates the TLR4–IL‐23–IL‐22 signaling pathway. (A) (i) Flow cytometric analysis of IL‐22 production in RORγt^+^ ILC3 cells. (ii) Proportion of IL‐22^+^ RORγt^+^ ILC3 cells among total RORγt^+^ ILC3 cells. (B) Plasma IL‐23 levels. (C) (i) Concentration of IL‐23 in the supernatant of bone marrow–derived macrophages (BMDMs). (ii) Concentration of IL‐23 in the supernatant of bone marrow–derived dendritic cells (BMDCs). ^#^DMSO versus ELPS; *DMSO versus ALPS. (D–F) Concentrations of IL‐23 (D), IL‐6 (E), and TNF‐α (F) in BMDMs supernatant after pretreatment with TAK242 (TLR4 antagonist, 10 μM), C29 (TLR2 antagonist, 50 μM), or CU‐CPT22 (TLR2/1 antagonist, 25 μM) for 1 h, followed by stimulation with ALPS or ELPS (100 ng/mL) for 24 h. (G) Immunofluorescent staining of TLR2 and TLR4 in BMDMs after incubation with ALPS or ELPS (1 μg/mL) for 7 h (scale bar, 20 μm). Shown are representative results from three replicates. (H) TLR4 signaling activity of ALPS was evaluated using HEK‐Blue™ reporter cells expressing mouse TLR4 (mTLR4). (I) Plasma IL‐23 levels in *Tlr4* ^−*/*−^ mice. (J) Plasma IL‐22 levels in *Tlr4* ^−*/*−^ mice. MOD: Model group, the high‐fat diet group. ALPS: *A. muciniphila* LPS group, ALPS‐treated high‐fat diet group. ALPS (In vitro): *A. muciniphila* LPS‐treated cells group, ELPS (In vitro): *E. coli* LPS‐treated cells group. ^ns^ *p* > 0.05, *p < 0.05, **p < 0.01, ***p < 0.001, ****p < 0.0001. ^#^ *p* < 0.05, ^##^ *p* < 0.01, ^###^ *p* < 0.001, ^####^ *p* < 0.0001. Unpaired *t*‐test in A–F, and H–J. *n* values: (A) *n* = 4, (B) *n* = 10, (C–G) *n* = 3, (H) *n* = 5, (I–J) *n* = 3–8.

Cytokines such as IL‐23, IL‐1β, IL‐6, and TNF‐α, produced by myeloid cells, are known to stimulate IL‐22 production in immune cells. In HFD‐fed mice, ALPS treatment increased IL‐23 levels in both plasma and ileum, while reducing IL‐1β, IL‐6, and TNF‐α concentrations (Figures 4, 5, and S12O–Q). In vitro assays further confirmed that ALPS robustly induces IL‐23 production (Figure 5C). IL‐22 in the gut is primarily secreted by innate lymphoid cells type 3 (ILC3s). Flow cytometry analysis revealed that ALPS significantly increased the proportion of IL‐22^+^ ILC3 in both the lamina propria (LP) and mesenteric lymph nodes (MLN) (Figure 5A). These findings suggest that IL‐23 is a key inducer of IL‐22 production in ALPS‐treated mice.

To determine the receptor specificity of ALPS, bone marrow–derived macrophages (BMDMs) were pretreated with Toll‐like receptor (TLR) antagonists, including TAK242 (TLR4), C29 (TLR2), and CU‐CPT22 (TLR2/1). Pretreatment with the TAK242 significantly suppressed ALPS‐induced production of IL‐6, IL‐23, and TNF‐α (Figure 5D–F). Western blot analysis and immunofluorescence staining confirmed activation of TLR4‐related signaling pathways (Figures 5G and S15), while TLR2 signaling showed relatively weak activation. ALPS also induced a TLR4‐mediated response in HEK‐Blue^TM^ reporter cells expressing TLR4 (Figure 5H). ALPS activated the maximal response at a dose of 1000 ng/mL, which is higher than the dose required for ELPS (500 ng/mL). These data collectively indicate that ALPS elicits a moderate immune response, primarily via TLR4 activation. To further validate the involvement of the TLR4–IL‐23–IL‐22 axis, ALPS was administered to *Tlr4* ^*−/−*^ mice. ALPS significantly increased IL‐22 and IL‐23 levels in *Tlr4* ^*+/−*^ mice and wild‐type (WT) mice, but not in *Tlr4* ^*−/−*^ mice (Figure 5I,J). These results demonstrate that ALPS induces IL‐22 production in immune cells through a TLR4‐dependent manner.

### An altered gut microbiota contributes to antiobesity effects of ALPS

**Figure 6:**
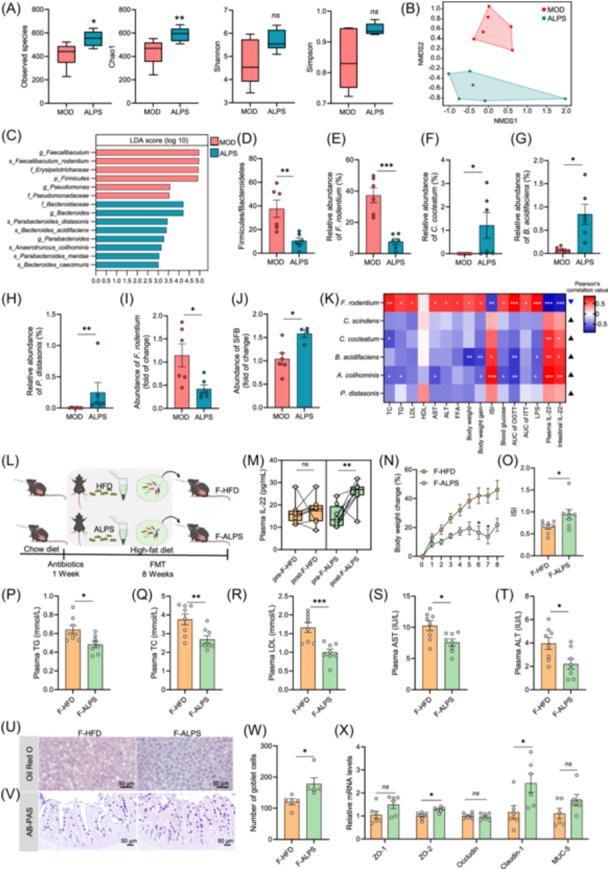
Alteration of gut microbiota by *Akkermansia muciniphila* LPS (ALPS) and the protective effect of ALPS‐induced gut microbiota in diet‐induced obese mice. (A) α diversity index. (B) Nonmetric multidimensional scaling (NMDS). (C) Linear discriminant analysis effect size (LEfSe) analysis. (D) Firmicutes/Bacteroidetes ratio. (E–H) Relative abundances of *Faecalibaculum rodentium, Clostridium cocleatum, Bacteroides acidifaciens*, and *Parabacteroides distasonis*. (I) Abundance of *F. rodentium*. (J) Abundance of Segmented Filamentous Bacteria (SFB) in the caecum, measured by qPCR (fold change). (K) Pearson correlation analysis of bacterial abundance (from 16S rRNA sequencing) with serum biochemical parameters and IL‐22 levels. Blue: negative correlation; red: positive correlation. Black equilateral triangles indicate bacteria significantly enriched in the ALPS group; inverted solid blue triangles indicate significantly decreased abundance. Pearson's *r* = 1 indicates a perfect positive linear correlation, *r* = −1 indicates perfect negative linear correlation, *r* = 0 indicates no linear correlation. (L) Fecal microbiota transplantation (FMT) experimental design showing treatment groups and durations. MOD: Model group, the high‐fat diet group. ALPS: *A. muciniphila* LPS group, ALPS‐treated high‐fat diet group. F‐HFD: The group of mice that received the feces of mice that were induced by a high‐fat diet. F‐ALPS: The group of mice that received the feces of mice that were treated by ALPS. (M) Plasma IL‐22 levels. (N) Body weight changes over time. (O) Plasma insulin sensitivity index (ISI). (P) Plasma triglyceride (TG). (Q) Plasma total cholesterol (TC). (R) Plasma low‐density lipoprotein (LDL). (S) Plasma aspartate aminotransferase (AST). (T) Plasma alanine aminotransferase (ALT). (U) Representative Oil Red O staining of liver sections (scale bar, 50 μm). (V) Representative AB‐PAS staining images of pathological ileum sections (scale bar, 50 μm). (W) Number of goblet cells in the ileum. (X) Relative mRNA levels of ZO‐1, ZO‐2, Occludin, Claudin‐1, and MUC‐5 in the ileum. ^ns^ *p* > 0.05, *p < 0.05, **p < 0.01, ***p < 0.001. Welch's *t*‐test in G–H, and J; unpaired *t‐*test in A, D–J, and W–X; Mann–Whitney *U* test in M, O–T. Two‐way ANOVA (Sidak) in N. *n* values: (A–I, X) *n* = 6, (L) *n* = 4–6, (M–T) *n* = 8, (U–W) *n* = 5.

Previous research has demonstrated that IL‐22 influences the gut microbiome through the upregulation of antimicrobial peptide (AMP) secretion, mucin production, and fucosylation by IECs [26, 40, 41]. To investigate the impact of ALPS on the gut microbiota in DIO mice, we analyzed the gut bacterial community. ALPS treatment enhanced bacterial species richness and α‐diversity (Figure 6A). Nonmetric multidimensional scaling (NMDS) and linear discriminant effect size (LEfSe) analyses revealed distinct intestinal bacterial communities between the ALPS‐treated group and the untreated model group (Figure 6B,C). Specifically, ALPS significantly reduced the ratio of Firmicutes/Bacteroidetes and the relative abundance of *Faecalibaculum rodentium* (Frod) (Figure 6D,E), while increasing the relative abundance of *Clostridium cocleatum*, *Bacteroides acidifaciens*, and *Parabacteroides distasonis* (Figure 6F–H). These bacterial species are associated with improved glucose and lipid metabolism, as previously report [42, 43, 44, 45]. Segmented filamentous bacterium (SFB), which induces IL‐22 production and occupies the same ecological niche as Frod, has been shown to alleviate metabolic disorders and enhance pathogen defense [46]. In this study, we confirmed that ALPS treatment increased SFB abundance and reduced Frod levels in the gut through qPCR analysis (Figure 6I,J). Additionally, ALPS enhanced the levels of acetic acid and butyrate in the gut (Figure S16), metabolites known to promote IL‐22 production [25].

To determine whether ALPS‐induced alterations in the gut microbiota contribute to protection against diet‐induced metabolic disorders, we performed fecal microbiota transplantation (FMT) experiments. A colony formation experiment verified the effective clearance of intestinal microorganisms in mouse feces (Figure S17S–T). Fecal microbiota from ALPS‐treated or untreated DIO mice were transferred to HFD‐fed and antibiotic‐treated mice (F‐ALPS or F‐HFD) (Figure 6L). The F‐ALPS group exhibited improved body weight gain, glucose and lipid metabolism, and reduced hepatic steatosis, along with significantly elevated plasma IL‐22 levels (Figures 6M–T and S17A–L). F‐ALPS mice also displayed enhanced intestinal barrier integrity and reduced inflammation (Figures 6U–X and S17M–R). These results indicate that ALPS‐modulated gut microbiota confer metabolic benefits to the host.

Finally, we assessed the safety of ALPS in mice (Figure S18). A single dose of 10 mg/kg led to nearly 40% mortality in the ELPS‐treated group, whereas no deaths occurred in the ALPS‐treated group at the same dose (Figure S18B,C). Furthermore, ALPS caused minimal damage to gut barrier function and induced a weaker inflammatory response compared to ELPS (Figure S18D–M). In addition, the antiobesity effects of ALPS and ELPS were compared at the same dosage of 0.2 mg/kg. As a result, ALPS and ELPS showed a similar effect in reducing body weight gain, but ALPS produced better efficacy than ELPS in decreasing the levels of hyperglycemia, hyperlipidemia, and IL‐6, and increasing the levels of IL‐22 and IL‐23 (Figure S19B–I). The antiobesity effects of ALPS, in combined with its favorable safety profile, support application of ALPS in alleviating the metabolic disorder diseases.

## DISCUSSION

Gut bacterial LPS plays a crucial role in maintaining and regulating immune homeostasis by inducing TLR‐mediated innate immune responses. For instance, LPS derived from gut symbiotic bacteria such as *Bacteroides thetaiotaomicron*, *B. vulgatus*, *Alcaligenes faecalis*, and *Veillonella parvula*, induces weak to moderate immune‐inflammatory or antagonistic responses, contributing to immune synergy or tolerance in the host [22, 47, 48, 49]. *A. muciniphila* has emerged as a promising candidate for next‐generation probiotics due to its therapeutic functions in treating obesity, hyperglycemia, atherosclerosis, and colitis [50, 51]. Characterizing the chemical properties of *A. muciniphila*‐derived LPS is essential for assessing its safety and uncovering its immunoregulatory mechanisms. In a recent work, Garcia‐Vello and colleagues characterized the chemical structure of LPS derived from a European *A. muciniphila* strain (Euro‐ALPS, ATCC BAA‐835), cultured in a synthetic medium containing N‐acetylglucosamine [31]. The lipid A moieties in Euro‐ALPS were identified as tetra‐acylated, penta‐acylated, or hexa‐acylated, and *mono*‐/*bis‐*phosphorylated. The core oligosaccharides were determined as a hexadecasaccharide (OS_1deAc_) and an undecasaccharide (OS_2deAc_), containing three 3‐deoxy‐d‐manno‐2‐oct‐ulopyranosonic acid (Kdo) moieties. The hexa‐acylated and *bis*‐phosphorylated lipid A moieties were predominant in Euro‐ALPS. In this study, we examined the chemical characteristics of LPS from a Chinese strain of *A. muciniphila*. (ALPS). ALPS was identified as a rough‐type LPS, primarily composed of the tetra‐acylated and *mono*‐phosphorylated lipid A. The oligosaccharide domains of ALPS consists of seven saccharide chains including two nonasaccharide chains (OS1–OS2), two decasaccharide chains (OS3–OS4), two tridecasaccharide chains (OS5–OS6), and one hexadecasaccharide (OS7). Genome comparison between strains HW07 and ATCC BAA‐835 reveals a 99.96% ANI similarity, between HW07 and ATCC BAA‐835, including the same gene cluster for lipid A and potential genes for oligosaccharide biosynthesis (Figure S20). It has been well documented that the metabolic products of a bacterium can vary depending on culture conditions [52, 53, 54]. The discrepancies observed in the lipid A and oligosaccharides distribution patterns between our findings and earlier reports could be attributed to differences in culturing parameters or the medium used. Given the lack of a homologous *lpxM* gene, the presence of hexa‐acylated LPS in the ATCC BAA‐835 strain further verification and its biosynthesis mechanisms deserve deep investigation.

The degree of acylation on lipid A influences LPS‐induced immune responses via TLR4 binding [20, 23]. For instance, smooth‐type LPS derived from pathogenic bacteria, such as the toxic LPS from *E. coli*, contains lipid A moieties with six or seven acyl chains and two phosphate groups, potentiating strong inflammatory responses. Structural studies on *E. coli* LPS binding to the TLR4/MD‐2 complex have elucidated mechanisms underlying differential immune activation [55]. Binding analysis revealed that five of the six acyl chains on lipid A of *E. coli* interact with a hydrophobic domain of MD2 in one TLR4–MD2 heterodimer, while the sixth acyl chain binds to another heterodimer. This interaction strongly activates the TLR4‐mediated signaling pathway. LPS with fewer than six acyl chains is unable to cross‐link two TLR4–MD2 heterodimers, leading to weak to moderate immune responses [56, 57]. In comparison to the strong inflammatory responses elicited by *E. coli* LPS, ALPS induce moderate immune responses primarily through activation of the TLR4 signaling pathway. Our current study highlights significant differences in immune activity between ALPS and Euro‐ALPS. Previous reports showed that intraperitoneal administration of Euro‐ALPS at 0.3 mg/kg induced increased expression of proinflammatory cytokines (MCP1 and IL‐1β) and anti‐inflammatory cytokine IL‐10 by activating both TLR4 and TLR2 signaling in mice. In this study, administration of a single 10 mg/kg dose of ALPS did not influence cytokine levels (TNF‐α, IL‐6, and IL‐1β) or survival rate compared to *E. coli* LPS. These discrepancies in immune activity are likely attributed to differences in lipid A moiety between ALPS and Euro‐ALPS.

In this study, we demonstrated that LPS derived from *A. muciniphila* strain HW07 mediates an IL‐22‐dependent antiobesity effect. ALPS activates the TLR4–IL‐23–IL‐22 axis, stimulating group 3 innate lymphoid cells (ILC3s) to produce IL‐22. The IL‐23/IL‐22 axis plays a critical role in regulating immune responses and balancing protective host defense against pathological inflammation [58]. IL‐23, primarily secreted by antigen‐presenting cells such as dendritic cells and macrophages, acts as a key regulator by activating IL‐22‐producing T helper 17 (Th17) cells or ILC3s. Intestinal IL‐22 is crucial for maintaining gut barrier integrity and mitigating metabolic disorders. Upon binding to the IL‐22 receptor complex (IL‐22R1/IL‐10R2), IL‐22 triggers JAK‐STAT signaling, leading to STAT3 phosphorylation [59]. Phosphorylated STAT3 forms dimers, translocating into the nucleus to initiate transcription of antimicrobial peptides, regenerating islet‐derived factors [60]. Additionally, pathways such as MAPK and PI3K/Akt may be activated, influencing cellular proliferation and differentiation [61]. Recent studies indicate that exogenous IL‐22 administration and inulin supplementation ameliorate metabolic health in HFD‐fed mice via an IL‐22‐dependent manner [25, 29]. We observed that ALPS significantly modulated the intestinal microbiota, increasing the relative abundance of *Clostridium cocleatum*, *Bacteroides acidifaciens*, and *Parabacteroides distasonis*, as well as Segmented filamentous bacterium (SFB) and the levels of acetate and butyrate levels. The flagellar protein of SFB promotes the expression of IL‐22 in Th17 cells [46]. SCFAs have the ability to induce the IL‐22 by binding to G‐protein receptor 41(GPR41) and inhibiting the activity of histone deacetylase (HDAC) [25].

Recent studies have highlighted some adverse effects of *A. muciniphila* in other diseases conditions. For instance, *A. muciniphila* induces an elevated IgE response, exacerbating food allergies in mice lacking dietary fiber [62]. Additionally, *A. muciniphila* colonization contributes to colitis in *IL‐10* ^*−/−*^ mice [63], and the metabolite succinate generated by *A. muciniphila* can enhance the virulence of *C. rodentium* [64]. These adverse effects of *A. muciniphila* may be associated with uncontrolled overgrowth in specific disease contexts. Besides hypo‐acylated rough‐type LPS, the outer membrane‐associated protein Amuc_1100, phosphatidylethanolamine have been confirmed as bioactive components in regulating obesity and immunity. Therefore, a pasteurized form of *A. muciniphila* containing these bioactive molecules, could be superior to its live counterpart as food supplement or therapeutic agent, considering safety concerns.

## CONCLUSION

The current study significantly enhances our understanding of *A. muciniphila* LPS as a critical biologically active compound linking to its probiotic properties. Moreover, it is indicated that distinctions in chemical structure and immunity activity of LPS containing in the products of *A. muciniphila* deserve attention. The TLR4–IL‐23–IL‐22 signaling axis demonstrated in the current work underscores a tightly orchestrated interaction between gut microbiota and host immunity and metabolism. The demonstrated antiobesity effect and immune‐regulatory mechanism support *A. muciniphila* LPS as a promising therapeutic agent for treating metabolic disorders including obesity and type 2 diabetes mellitus.

## METHODS

### Extraction of LPS from *A. muciniphila*

*A. muciniphila* HW07 from a Chinese healthy adult was cultured at 37°C for 5 days in anaerobic modified brain‐heart infusion (BHI) broth with addition of mucin [65]. The *A. muciniphila* LPS was extracted for two times on the basis of an early reported method [22]. In brief, after centrifugation at 10,000 rpm for 30 min, bacteria were collected, washed with 0.15 mol/L NaCl solution, and lyophilized. The resulting bacterial cells (40.4 g) were extracted with phenol/water to yield crude LPS that was removed from nucleic acids, proteins, and phospholipids by treatment of DNase, RNase, proteases, and repeated washing with CH_3_Cl─MeOH and CH_3_Cl─MeOH─H_2_O. The further purification was conducted with Sephadex G‐50 gel filtration chromatography, eluting with distilled water. The protein content of ALPS was determined by BCA protein quantitative kit. The nature and purity of ALPS were detected by SDS‐PAGE and silver staining [66], with LPS from *Escherichia coli* O111:B4 (L2630‐100MG; ELPS; Sigma) as a control. The average content of LPS in *A. muciniphila* is calculated to be 54.6 ± 3.1 mg/g (*n* = 3) according to their yields.

### Chemical analysis of ALPS

The total fatty acids and monosaccharides were obtained from the dried ALPS (1.0 mg) by acidic methanolysis [67]. The hexane partition containing fatty acids was analyzed using an Agilent 7890A gas chromatography‐mass spectrometer with the following temperature program: 150°C for 3 min; 150°C to 280°C by 3°C/min, and 280°C for 10 min. The methanol partition was dried and further analyzed for the monosaccharide composition by high‐performance anion‐exchange chromatography (HPAEC) (Dionex ICS‐5000+) coupled with the electro‐chemical detector (ECD) and a Dionex CarboPac PA20 analytical column (150 × 3.0 mm), with an isocratic elution of 2 mM NaOH (30°C, 0.4 mL/min), in comparing with the corresponding monosaccharide standards. The heptose (Hep*p*) in the ALPS was confirmed by LC‐MS analysis. The 3‐deoxy‐D‐*manno*‐2‐oct‐ulopyranosonic acid in the ALPS were detected using 100 mM sodium acetate in 20 mM aqueous NaOH as eluent (30°C, 0.5 mL/min).

### Characterization of the oligosaccharides and lipid A moieties from ALPS

The oligosaccharides and lipid A were obtained from ALPS according to early reports [22]. The lipid A was obtained from ALPS by mild acid hydrolysis. Briefly, ALPS (200 mg) was hydrolyzed with 12.5 mM acetic acid buffer (27.0 mL, pH = 4.5) containing SDS at a concentration of 1.0 mg/mL at 100°C for 90 min. After reaction, a chloroform/methanol/water solution (100:30:30, v/v/v) was added. The chloroform/methanol extract collected by centrifugation was dried to give crude lipid A. The main lipid A species were further purified by preparative thin‐layer chromatography developing with CHCl_3_/MeOH/H_2_O (300:120:20) and stained with distilled water from the crude lipid A. The deacylated oligosaccharides were obtained from ALPS by reduction and alkaline hydrolysis. In brief, the dried ALPS (100 mg) was dissolved with hydrazine solution at a concentration of 20 mg/mL and stirred at 37°C for 1.5 h. After reaction, the O‐deacylated product was poured into ice‐cold acetone (20 mL), following by centrifugation and lyophilization. Subsequently, the obtained O‐deacylated product was heated with 4 M KOH at 120°C for 16 h, cooled, and allowed to precipitate. The resulting aqueous phase was first extracted with dichloromethane to remove fatty acids, then adjusted to acidity (pH = 6) followed by dialysis with a molecular mass cutoff of 500 Da and lyophilization to give deacylated oligosaccharides. The ESI‐MS data of the deacylated and lipid A were recorded with an Agilent Accurate‐Mass‐Q‐TOF LC/MS 6520 in the negative ion mode, respectively. For HPLC‐ESI‐MS analysis with in‐source fragmentation mode, the oligosaccharides were dissolved in water and subjected to analysis using a Shim‐pack GIST C18‐AQ column (250 × 4.6 mm; 5 μm; SHIMADZU) with the flow rate at 0.3 mL/min and the column temperature at 45°C in the scanning range of *m/z* 60–2500. A gradient elution beginning with mobile phase A (0.01% formic acid) for 3 min, then with an increase of mobile phase B (acetonitrile) to 100% (v/v) over 10 min, following with 100% B for 5 min and a decrease to 100% A in 8 min, was applied. The lipid A was dissolved in CHCl_3_/MeOH (2:1, v/v) and loaded on the Eclipse Plus C18 column (50 × 2.1 mm, 1.8 μm; Agilent) for HPLC‐ESI‐MS^2^ analysis in the range of *m/z* 60–2600. A gradient elution beginning with solvent A (80% MeOH–H_2_O solution with 200 mM ammonium hydroxide) for 2 min, then with an increase of solvent B (*i*‐propanol with 200 mM ammonium hydroxide) to 95% (v/v) over 15 min, following with 95% B for 4 min and a decrease to 10% A in 7 min, was applied. The deacylated oligosaccharides were further separated by a combination of ion exchange chromatography and size exclusion chromatography, affording the oligosaccharide OS7. For structural assignments of oligosaccharide 1D and 2D NMR spectra were recorded on a Bruker AVANCE‐500 spectrometer equipped with a cryoprobe. The solvent employed was D2O.

### Cell culture and in vitro assays

RAW264.7 cells purchased from ATCC was cultured with DMEM (6016121; Dakewe) supplemented with 10% FBS (RY‐F81‐05; Royacel) and 1% penicillin and streptomycin (MA0110; Meilunbio). The bone marrow cells were cultured in complete DMEM supplemented with GM‐CSF (10 ng/mL) (HY‐P7361; MedChemExpress) for BMDMs differentiation, and cultured in complete RPMI‐1640 medium supplemented with GM‐CSF (20 ng/mL) and IL‐4 (1 ng/mL) (CK15; Novoprotein) for BMDC differentiation. Before the bioactivity assay, BMDM and BMDC cells were allowed to grow for 7–10 days, with additional feeding of 20–40 ng/ml GM‐CSF on third day.

For TLR4 activation detection, the HEK‐Blue^TM^ TLR4 cells that stably transfected with a secreted embryonic alkaline phosphatase (SEAP) reporter gene, were cultured based on manufacturer's specifications. Cells were seeded into 96‐well plates and co‐treated with LPS/ALPS and HEK‐Blue^TM^ detection medium containing a SEAP‐specific chromogenic substrate. Following a 6‐h incubation (37°C, 5% CO₂), SEAP enzymatic activity that reflects the TLR4‐induced NF‐κB activation was quantified spectrophotometrically at 650 nm.

For inhibitor assay, BMDMs were seeded into 24‐well plates and preincubated with 10 μM TAK242 (HY‐11109; MedChemExpress), 50 μM C29 (HY‐100461; MedChemExpress), or 25 μM CU‐CPT22 (T15020; TargetMol) for 1 h, respectively. The supernatant was then removed and replaced with fresh medium, following by addition of ELPS or ALPS at a final concentration of 100 ng/mL and incubation for 24 h.

### Immunofluorescence staining of TLR4 and TLR2

BMDMs seeded on a glass plate were incubated with 1 μg/mL ELPS or ALPS for 7 h, then washed and fixed in 4% paraformaldehyde for 1 h. After permeabilization with cell permeabilization buffer (P0096; Beyotime) for 1 h, cells were cleaned twice with cold PBS (pH 7.4), then incubated with anti‐TLR2 (23333; Zen Bio), anti‐TLR4 (Proteintech; 66350‐1; Proteintech) overnight at 4°C. After washing with 1× PBS buffer (pH 7.4), cells were added with 5% goat serum added, following with Alexa Fluor 488 goat anti‐mouse IgG (A23210; Abbkine) or Alexa Fluor 546 Affini Pure goat anti‐rabbit IgG secondary antibodies (A‐11035; Invitrogen) for another 1 h. Fluorescence was visualized under a Leica SP8 confocal microscope.

### Mice

6–8 weeks male C57BL/6J, DIO mice were purchased from GemPharmatech Co., Ltd. *Tlr4* ^*−/−*^ mice (Cyagen Biosciences) were used for breeding. The target alleles were identified by PCR and DNA sequencing. The genotyping primers used were: F1: 5′‐GGAATCCATGCACTATAAGACA‐3′, R1: 5′‐CCAGTAGCTTTAGGAATATGGTAG‐3′, R2: 5′‐GGTAACTAATTGCTGTGCTATTG‐3′. All animals were housed with equal durations of light and dark under specific pathogen‐free (SPF) conditions. The chow diet (13.5% calories from fat; HFK; 2032) and a HFD (60 kcal% fat as indicated, New Brunswick; D12492) were used for feeding. Experiments were performed after prior approval by the Ethics Committee of Institute of Microbiology (IMCAS2024126), Chinese Academy of Sciences.

For metabolic syndrome assay, 20 mice receiving 8 weeks of HFD feeding were divided into two groups (*n* = 10 each). Mice were intraperitoneally injected with ALPS at the dose of 0.2 mg/kg (ALPS group) once 2 days or an equal volume (0.1 mL) normal saline (MOD group).

For acute endotoxicity assay, C57BL/6J mice fed with chow diet were divided into the ELPS, ALPS and saline groups (*n* = 6 each). Mice were intraperitoneally administrated with a single dose of LPS from *Escherichia coli* O111:B4 (ELPS, 10 mg/kg, L2630; Sigma) and ALPS (10 mg/kg), respectively. The survival of the mice was observed and killed 24 h after administration, and plasma and intestinal tissue were collected for further analysis.

For anti‐IL‐22 antibody neutralization assay, DIO mice were subset into four groups (*n* = 6 each) after 8 weeks of HFD feeding, including the vehicle‐treated HFD‐fed group (HFD), the ALPS‐treated HFD‐fed group (HFD+ALPS), the αIL‐22‐treated HFD‐fed group (HFD+αIL‐22), and the αIL‐22 and ALPS‐treated HFD‐fed group (HFD+αIL‐22+ALPS). Mice were intraperitoneally treated with IL‐22‐neutralizing antibody (5 μg, 500‐P223; PeproTech) once 2 days for 5 weeks. ALPS (0.2 mg/kg) was intraperitoneally administrated once 2 days during the course of assay.

For *Tlr4* ^*−/−*^ mice experiments, 8‐weeks male of *Tlr4* ^*−/−*^ (*n* = 3 for vehicle group; *n* = 4 for ALPS group), *Tlr4* ^*+/−*^ (*n* = 8 for each group), and wide type WT, (*n* = 7 for each group) mice were intraperitoneally administrated with ALPS at the dose of 0.2 mg/kg and equal volume (0.1 mL) of normal saline once 2 days, respectively. Plasma was collected a week later for ELISA analysis.

For comparison of antimetabolic syndrome effects, C57BL/6J mice that fed with HFD were intraperitoneally administrated with ALPS (ALPS group, *n* = 9), ELPS (ELPS group, *n* = 9) at the dose of 0.2 mg/kg once 2 days, or equal volume (0.1 mL) normal saline (MOD group) for 5 weeks.

The body weight and blood glucose were measured weekly. Fecal samples were collected 2 weeks before the end of the experiment and stored at −80°C. OGTT and ITT also tested 2 weeks before the end of the experiment. The mice were killed and the plasma, organs, fat, and intestinal tissues of the mice were collected and stored at −80°C for analysis.

### Biochemical and immunological assays

Levels of cytokines IL‐6 (1210602; Dakewe), IL‐1β (RK04878; Abclonal), IL‐10 (SEKM‐0007; Solarbio), IL‐22 (1212202; Dakewe), IL‐23 (KE10068; Proteintech), TNF‐α (RK04875; Abclonal) in cell supernatants, plasma, and ileum were determined by Elisa kits, and levels of insulin, HbA1c, LPS, ox‐LDL, Hs‐CRP, AST, ALT, T‐CHO, TG, LDL‐C, and FFA in plasma were determined using commercial kits. Insulin sensitivity index = (1000 × fasting blood insulin (mU/L) × fasting blood glucose (mg/dL))^*−*1^.

### Quantitative real‐time PCR

Total RNA from cell, ileum, and liver were extracted using the TRIzol (10606ES60; Yeasen). cDNA was obtained using the reverse transcription cDNA synthesis kit (KR116‐02; Tiangen). 2×SuperFast Universal SYBR Master Mix (CW3888H; CWBIO) was used for qPCR. Glyceraldehyde 3‐phosphate dehydrogenase or 16S ribosomal RNA (rRNA) were used to normalize with $2^{-∆∆C_{t}}$. All primer sequences are shown in Table S2.

### Histological analysis

The liver, adipose tissues, and intestinal tissues were isolated and fixed in 4% paraformaldehyde solution. The livers were stained with Sirius red and Oil Red O, and the adipose tissues were stained with H&E. The intestinal tissues were treated with H&E and AB‐PAS for staining. The lesion area, the number of goblet cells, the relative area of Oil Red O, the collagen volume fraction (VCF), the crypt and TA heights were measured using ImageJ software. Quantitative adipocyte size analysis using ImageJ: (1) Image Processing: Import calibrated adipose tissue histological sections into ImageJ. (2) Measurement setup: Apply the elliptical tool to trace adipocyte cross‐sections, excluding incomplete or overlapping cells. (3) Data acquisition: Execute analyze‐measure to extract parameters (area, perimeter, major/minor axes). (4) Statistical analysis: Calculate mean cross‐sectional area and generate frequency distribution histograms. Finally, the data is normalized.

### Immunofluorescence analysis

Fresh intestine tissues were immediately fixed in 4% paraformaldehyde solution for 24 h and further blocked with 5% bovine serum albumin for 30 min. Then, the 3–5 μm thick slides were incubated overnight at 4°C with primary antibody (the anti‐IL‐22 rabbit pAb; GB11259‐100; Servicebio), following with the secondary antibody (the goat anti‐rabbit IgG‐Cy3; GB21303; Servicebio) for 50 min at room temperature. ImageJ software was used to quantify the images.

### Western blot

The BMDMs were incubated with ALPS/ELPS for 24 h and ileal tissue of ALPS‐treated mice were homogenized in the cold RIPA lysis buffer (C5029; Bioss). The protein was first separated by SDS‐PAGE and then transferred to polyvinylidene fluoride (PVDF; 1620184; Bio‐Rad) membrane. Transmembrane efficiency was verified using the Ponceau Staining Solution (HY‐K1109; MedChemExpress). The membranes were closed with 5% nonfat milk at room temperature for 1 h, which was followed by incubation overnight at 4°C with different antibodies: Rabbit anti‐TLR4 (14358; Cell Signaling Technology), anti‐TLR2 (ab209216; Abcam), anti‐IL‐22 (A6216; ABclonal), anti‐Claudin1 (343203; Zen Bio), anti‐Occludin (502601; Zen Bio) and β‐actin (HY‐P80438; MedChemExpress). Finally, HRP‐linked goat anti‐rabbit IgG (BS13278; Bioworld) secondary antibody or goat anti‐mouse IgG (A0216; Beyotime) was added for 1‐h incubation. Proteins were visualized using ECL Plus Western Blot assay (BL520A; Biosharp). β‐Actin was used to normalize the expression of IL‐22.

### Gut microbiota analysis

Caecal DNA was extracted, sequenced and analyzed on an Illumina NovaSeq sequencing platform as described in our early work [45]. Primers for the 16S rRNA variable region 3‐4 (V3‐V4) were 338 F (5′‐ACTCCTACGGGAGGCAGCA‐3′) and 806 R (5′‐GGACTACHVGGGTWTCTAAT‐3′). The raw 16S rDNA data is available on gcMeta at https://nmdc.cn/. with project ID NMDC40051824‐NMDC400518235. To determine the level of short‐chain fatty acids in the gut [68], the lyophilized fecal samples acidified with 50% sulfuric acid was extracted with ether. The ether extracts obtained by centrifugation at 5000 rpm for 15 min were put forward to GC‐MS analysis.

### Flow cytometry analysis

The intestinal pieces collected after removing Peyer's patches were incubated at 37°C in 5 mM EDTA Ca^2+^ and Mg^2+^ free RPMI‐1640 medium for 20 min and then washed with PBS to remove epithelial layers and mucus. The fragments were cut thoroughly and digested twice at 37°C for 30 min with 2% RPMI‐1640 (vol/vol) FBS, 1.0 mg/mL collagenase II and III (WBC‐LS004176 and WBC‐LS004182; Worthington), and 0.2 mg/mL DNase I (64002860; SCR). After centrifugation, the intestinal lamina propria (LP) cells were passed through the 70 μM cell filter and washed twice with PBS for staining at 4°C.

After culturing with a cell activation cocktail (423304; BioLegend) for 6 h, the cells were stained with anti‐CD16/32 (553141; BD) for 5 min and then treated with zombie (423106; BioLegend) for 15 min on the ice. In the following incubation, the cells were surface stained with a mixture of anti‐CD3‐FITC (100204; BioLegend), anti‐CD45‐BV510 (BD; 563891), anti‐CD127‐BV711 (BD; 565490), anti‐Gr1‐PE‐cy7 (108416; BioLegend) for 30 min at 4°C. Finally, the cells were fixed and permeabilized with transcription factor buffer set (562574; BD), followed by staining with anti‐RORγt‐BV650 (564722; BD) and anti‐IL‐22‐AF647 (516409; BioLegend). The stained cells were passed through the 70 μm cell filter and washed twice with staining buffer. The cells collected on a FACS Aria II flow cytometer (LSRFortessa SORP; Becton Dickinson) were analyzed by FlowJo. The analysis was conducted based on the flow cytometry gating strategies (Figure S21).

### Fecal microbiota transplantation

The FMT experiment was conducted as referred to an early study [69]. Mice were treated with a combination of broad‐spectrum antibiotics in drinking water containing neomycin (1 g/L), ampicillin (1 g/L), metronidazole (0.5 g/L), and vancomycin (0.5 g/L) for 7 days to deplete intestinal microorganisms. The pseudo‐germ‐free mice fed with HFD were randomly divided into two groups: F‐HFD and F‐ALPS group. The HFD‐fed mice receiving 8‐weeks treatment of ALPS were used as donors. After 4 weeks treatment, the fresh feces collected from the HFD‐ALPS group were weighted, re‐suspended at 100 mg/mL in PBS. Fresh feces from the HFD‐fed group were treated in the same manner. Mice were orally given with 0.2 mL of feces suspension daily for 8 weeks.

### Statistical analysis

Correlations between species and factors were analyzed using Pearson's correlation coefficient. Pearson's correlation analysis calculates the Pearson's *r*, *r* = 1 indicates a completely positive linear correlation, *r* = −1 indicates complete negative linear correlation, *r* = 0 indicates no linear correlation. The normality of the distribution was tested by the Kolmogorov–Smirnov test. Comparisons between the two groups are represented by Welch's *t*‐test, unpaired *t*‐test, and Mann–Whitney *U* test. Welch's *t*‐test required normality in both groups but did not require equal variances. The unpaired *t*‐test required both normality and equal variances. The Mann–Whitney *U* test required neither normality nor equal variances. One‐way ANOVA test (post‐event verification method: Tukey's HSD), Kruskal–Wallis test (post‐event verification method: Dunn's Test), and Welch's ANOVA test (post‐event verification method: Games‐Howell) were used for multiple comparisons. Two‐way ANOVA (post‐event verification method: Sidak) was used when processing multiple measurement data of the same subject. The data in the figure was expressed as mean ± SEM. GraphPad Prism 9.0 and ImageJ were used to analyzed all the data and showed as: ^ns^ *p* > 0.05, *p < 0.05, **p < 0.01, ***p < 0.001, ****p < 0.0001. ^#^ *p* < 0.05, ^##^ *p* < 0.01, ^###^ *p* < 0.001, ^####^ *p* < 0.0001.

## AUTHOR CONTRIBUTIONS

**Li Sun**: Data acquisition and analysis; writing—original draft; methodology; visualization. **Yuting Zhang**: Data acquisition and analysis; writing—original draft; methodology; visualization. **Wang Dong**: Data acquisition and analysis; writing—original draft; methodology. **Jingzu Sun**: Formal analysis; methodology. **Tao Wang**: Methodology. **Fei Shao**: Methodology. **Huanqin Dai**: Funding acquisition. **Wenzhao Wang**: Methodology. **Shuo Wang**: Methodology; formal analysis. **Tong Zhao**: Methodology. **Liangliang Wang**: Resources. **Chang Liu**: Resources. **Shuangjiang Liu**: Resources. **Hongwei Liu**: Supervision; funding acquisition; writing; review and editing the manuscript.

## CONFLICT OF INTEREST STATEMENT

The authors declare no conflicts of interest.

## ETHICS STATEMENT

The ethics application (IMCAS2024126) was approved by the Institute of Microbiology, Chinese Academy of Sciences of Research Ethics Committee.

## Supporting information


**Figure S1**: Fatty acid content of *A. muciniphila* HW07 R‐LPS, which was determined by GC‐MS analysis of related methyl esters derivatives.
**Figure S2**: Monosaccharide analysis of *A. muciniphila* LPS (ALPS).
**Figure S3**: TLC profile of the acetic acid hydrolysis products from ALPS.
**Figure S4**: HPLC‐ESI‐MS^2^ spectroscopic analysis of the lipid A of 1335.9328 in *A. muciniphila* LPS (ALPS).
**Figure S5**: HPLC‐ESI‐MS^2^ spectroscopic analysis of the lipid A of 1349.9494 in *A. muciniphila* LPS (ALPS).
**Figure S6**: HPLC‐ESI‐MS^2^ spectroscopic analysis of the lipid A of 1295.9026, 1309.9176, 1363.9468, 1375.9637, 1389.9736, 1403.9935, 1430.0039 in *A. muciniphila* LPS (ALPS).
**Figure S7**: Negative mode in‐source fragmentation‐based high‐performance liquid chromatography‐electrospray ionization‐mass spectrometry (in‐source fragmentation‐HPLC‐ESI‐MS) analysis of oligosaccharides from *A. muciniphila* LPS (ALPS).
**Figure S8**: Negative mode in‐source fragmentation‐based ESI‐MS spectrum and the proposed sugar sequence of oligosaccharides OS1‐OS7.
**Figure S9**: The ^1^H NMR (A) and ^13^C NMR (B) spectrum of oligosaccharide OS7 from *A. muciniphila* HW07.
**Figure S10:** *A. muciniphila* HW07 LPS chemical structure.
**Figure S11:** Immunological properties of *A. muciniphila* LPS (ALPS) in myeloid cells.
**Figure S12:** *A. muciniphila* LPS (ALPS) improved obesity and related glucose and lipid metabolism dysfunctions in DIO mice.
**Figure S13:** The RNA‐seq liver transcriptome analysis.
**Figure S14:** Neutralization of IL‐22 abrogated the hypoglycemic action of *A. muciniphila* LPS (ALPS).
**Figure S15:** *A. muciniphila* LPS (ALPS) mainly functions through the TLR4 signaling pathway.
**Figure S16:** *A. muciniphila* LPS (ALPS) promoted the production of short‐chain fatty acids (SCFAs) in DIO mice.
**Figure S17:** *A. muciniphila* LPS (ALPS)‐altered gut microbiota protected high fat diet‐induced metabolic disorders.
**Figure S18:** Comparison of acute endotoxemia of ELPS and ALPS in C57BL/6 J mice.
**Figure S19:** The effects of ELPS and ALPS in DIO mice.
**Figure S20:** ANI analysis between *A. muciniphila* HW07 and ATCC BBA‐835.
**Figure S21:** Flow cytometry gating strategies. Gating strategies for IL‐22^+^RORγt^+^ ILC3 cells.
**Table S1:** Analysis of LPS biosynthetic genes in the genome of *Akkermansia muciniphila*.
**Table S2:** Primers sequences in the experiments.

## Data Availability

The raw 16S rRNA gene amplicon sequencing data reported in this paper have been deposited in the Global Catalogue of Metagenomics platform (gcMeta), Chinese Academy of Sciences Initiative of Microbiome (https://nmdc.cn/) under project accession ID: NMDC10018704 (https://nmdc.cn/resource/genomics/project/detail/NMDC10018704). RNA‐seq data have been deposited in NCBI's NLM and can be accessed through BioProject ID: PRJNA1280525 (http://www.ncbi.nlm.nih.gov/bioproject/1280525). The data and scripts used are saved in GitHub https://github.com/Veinard05/imeta-2025-586. Supporting Information (figures, tables, graphical abstract, slides, videos, Chinese translated version, and update materials) may be found in the online DOI or iMeta Science https://www.imeta.science/.
