# Supplementary material for: Akkermansia muciniphila‐derived hypoacylated rough‐type lipopolysaccharides alleviate diet‐induced obesity via activation of TLR4−IL‐23−IL‐22 immune axis
Source: Imeta. 2025 Jul 17;4(5):e70066. doi: 10.1002/imt2.70066 (PMC12527988; doi:10.1002/imt2.70066)
Supplement: Supplementary file 1 — Figure S1: Fatty acid content of A. muciniphila HW07 R‐LPS, which was determined by GC‐MS analysis of related methyl esters derivatives. Figure S2: Monosaccharide analysis of A. muciniphila LPS (ALPS). Figure S3: TLC profile of the acetic acid hydrolysis products from ALPS. Figure S4: HPLC‐ESI‐MS2 spectroscopic analysis of the lipid A of 1335.9328 in A. muciniphila LPS (ALPS). Figure S5: HPLC‐ESI‐MS2 spectroscopic analysis of the lipid A of 1349.9494 in A. muciniphila LPS (ALPS). Figure S6: HPLC‐ESI‐MS2 spectroscopic analysis of the lipid A of 1295.9026, 1309.9176, 1363.9468, 1375.9637, 1389.9736, 1403.9935, 1430.0039 in A. muciniphila LPS (ALPS). Figure S7: Negative mode in‐source fragmentation‐based high‐performance liquid chromatography‐electrospray ionization‐mass spectrometry (in‐source fragmentation‐HPLC‐ESI‐MS) analysis of oligosaccharides from A. muciniphila LPS (ALPS). Figure S8: Negative mode in‐source fragmentation‐based ESI‐MS spectrum and the proposed sugar sequence of oligosaccharides OS1‐OS7. Figure S9: The 1H NMR (A) and 13C NMR (B) spectrum of oligosaccharide OS7 from A. muciniphila HW07. Figure S10: A. muciniphila HW07 LPS chemical structure. Figure S11: Immunological properties of A. muciniphila LPS (ALPS) in myeloid cells. Figure S12: A. muciniphila LPS (ALPS) improved obesity and related glucose and lipid metabolism dysfunctions in DIO mice. Figure S13: The RNA‐seq liver transcriptome analysis. Figure S14: Neutralization of IL‐22 abrogated the hypoglycemic action of A. muciniphila LPS (ALPS). Figure S15: A. muciniphila LPS (ALPS) mainly functions through the TLR4 signaling pathway. Figure S16: A. muciniphila LPS (ALPS) promoted the production of short‐chain fatty acids (SCFAs) in DIO mice. Figure S17: A. muciniphila LPS (ALPS)‐altered gut microbiota protected high fat diet‐induced metabolic disorders. Figure S18: Comparison of acute endotoxemia of ELPS and ALPS in C57BL/6 J mice. Figure S19: The effects of ELPS and ALPS in DIO mice. Figure S20: [file IMT2-4-e70066-s001.docx]

**Supporting information to**

***Akkermansia muciniphila*-derived hypoacylated rough-type lipopolysaccharides alleviate diet-induced obesity via activation of TLR4**−**IL-23**−**IL-22 immune axis**

Running title: *A. muciniphila* Hypoacylated LPS Activates TLR4−IL23−IL22 Against Obesity

Li Sun ^1, 2, #^, Yuting Zhang ^1, 2, #^, Wang Dong ^1, 2, #^, Jingzu Sun ^1, #^, Tao Wang ^1^, Fei Shao ^3^, Huanqin Dai ^1, 2^, Junjie Han ^1, 2^, Wenzhao Wang ^1^, Shuo Wang ^3^, Tong Zhao^4^, Liangliang Wang ^5^, Chang Liu ^6^, Shuangjiang Liu ^6^, Hongwei Liu ^1, 2, 5*^

^1^ State Key Laboratory of Microbial Diversity and Innovative Utilization, Institute of Microbiology, Chinese Academy of Sciences, Beijing, P. R. China.

^2^ Medical School, University of Chinese Academy of Sciences, Beijing, P. R. China.

^3^ CAS Key Laboratory of Pathogen Microbiology and Immunology, Institute of Microbiology, Chinese Academy of Sciences, Beijing, P. R. China.

^4^ Institution Center for Shared Technologies and Facilities, Institute of Microbiology, Chinese Academy of Sciences, Beijing, P. R. China

^5^ The Laboratory of Microbiome and Microecological Technology, Institute of Microbiology, Chinese Academy of Sciences, Beijing, P. R. China.

^6^ State Key Laboratory of Microbial Technology, Shandong University, Qingdao, P. R*.* China.

^#^ These authors contributed equally: Li Sun, Yuting Zhang, Wang Dong, Jingzu Sun,

* Corresponding: liuhw@im.ac.cn (Hongwei Liu)

**Supplemental Figure**

**Figure S1**. Fatty acid content of *A. mucinniphila* HW07 R-LPS, which was determined by GC-MS analysis of related methyl esters derivatives. Fatty acids of ALPS: a, C12:0 (3-OH); b, *i*-C14:0; c, C13:0 (3-OH); d, *i*-C15:0; e, C15:0; f, C14:0 (3-OH); g, C16:0; h, C15:0 (3-OH); i, C17:0; j, C16:0 (3-OH); k, C18:0; l, C17:0 (3-OH). Fatty acid standard: 1. C10:0, 2. C11:0, 3. C12:0, 4. C13:0, 5. C14:1, 6. C14:0, 7. C15:1, 8. C15:0, 9. C16:1, 10. C16:0, 11. C17:1, 12. C17:0, 13. C18:3, ω-3 , 14. C18:2, 15. C18:3, 16. C18:2T, 17. C18:1, 18. C18:0, 19. C20:5, 20. C20:4, 21. C20:3, 22. C20:2, 23. C20:1, 24. C20:0, 25. C14:0 (3-OH), 26. C15:0 (3-OH), 27. C16:0 (3-OH), 28. C18:0 (3-OH). The *iso*-terminal type of the aliphatic chain of fatty acid was represented by *i-*. Fatty acids of b, d, and l was determined by comparison of the GC-MS spectrum and calculation of retention index RI. RI = n × 100+100 × (Ti-Tn)/(Tn+1-Tn).

**Figure S2**. Monosaccharide analysis of *A. mucinniphila* LPS (ALPS). (A) HPAEC of monosaccharide composition of the acid hydrolysis product of ALPS and standard mixture. (1. Fuc; 2. GalN; 3. Rha; 4. GlcN; 5. Gal; 6. Glc; 7. Man; 8. Hep.) (B) MS spectrum of Hep. (C) HPAEC of acidic sugar composition of ALPS and standard of Kdo.

**Figure S3**.TLC profile of the acetic acid hydrolysis products from ALPS. TLC was developed with a mixture of CHCl_3_/MeOH/H_2_O (300:120:20) and stained with distilled water.

**Figure S4**. HPLC-ESI-MS^2^ spectroscopic analysis of the lipid A of 1335.9328 in *A. mucinniphila* LPS (ALPS). (A) MS/MS spectrometry of *m/z* 1335.9328. (B) A proposed fragmentation scheme of *m/z* 1335.9328.

**Figure S5**. HPLC-ESI-MS^2^ spectroscopic analysis of the lipid A of 1349.9494 in *A. mucinniphila* LPS (ALPS). (A) MS/MS spectrometry of *m/z* 1349.9494. (B) A proposed fragmentation scheme of *m/z* 1349.9494.

**Figure S6**. HPLC-ESI-MS^2^ spectroscopic analysis of the lipid A of 1295.9026, 1309.9176, 1363.9468, 1375.9637, 1389.9736, 1403.9935, 1430.0039 in *A. mucinniphila* LPS (ALPS). (A-G) *m/z* 1363.9468. (A) 1309.9176. (B) 1295.9026. (C) 1375.9637. (D) 1389.9736. (E) 1403.9935. (F) 1430.0039. (G) Chain length is indicated by the number in the circle on the acyl chain.

**Figure S7**. Negative mode in-source fragmentation-based high-performance liquid chromatography-electrospray ionization-mass spectrometry (in-source fragmentation-HPLC-ESI-MS) analysis of oligosaccharides from *A. mucinniphila* LPS (ALPS). (A) Total ion chromatogram of oligosaccharides from deacylated ALPS. (B) Negative mode ESI-MS spectrum of OS1-OS7. [M-2H]^2-^ ions responsible for OS1-OS7 were observed at *m/z* 869.76, 909.74, 950.29, 990.28, 1184.90, 1224.88, and 1622.51, respectively.

**Figure S8**. Negative mode in-source fragmentation-based ESI-MS spectrum and the proposed sugar sequence of oligosaccharides OS1-OS7. The glycan chains of OS from *A. muciniphila* HW07 presented as the symbology of mono-sugar units. The double charged ion at *m/z* 909.74 (A, OS2), 869.76(B, OS1), 950.29 (C, OS3), 990.28 (D, OS4), 1184.89 (E, OS5), 1224.88 (F, OS6), 1622.51 (G, OS7) were detected, respectively. The fragment ions at *m/z* 419, 499, 719, 859 and 939 are derived from the mono-charged molecular ions. “Hex”, hexose; “Hep”, heptose; “Fuc”, fucose; “HexN”, hexosamine; “*P*”, phosphate; “Kdo”, 3-deoxy-D-manno-2-oct-ulopyranosonic acid, respectively.

**Figure S9**. The ^1^H NMR (A) and ^13^C NMR (B) spectrum of oligosaccharide OS7 from *A. mucinniphila* HW07.

**Figure S10.** *A. muciniphila* HW07 LPS chemical structure. (A) The glycan chains of OS7 from *A. mucinniphila* HW07 strain presented as the symbology of mono-sugar units. (B) Multiplicity-edited ^1^H, ^13^C HSQC spectrum of oligosaccharide OS7 from *A. mucinniphila* HW07. (C) Zoom of the ^1^H, ^13^C HSQC spectrum of oligosaccharide OS7 from *A. mucinniphila* HW07*.*

**Figure S11.** Immunological properties of *A. mucinniphila* LPS (ALPS) in myeloid cells. (A) TNF-α concentration in cell supernatant of RAW 264.7. (B) IL-6 concentration in cell supernatant of Raw 264.7. (C) TNF-α concentration in cell supernatant of BMDMs. (D) IL-6 concentration in cell supernatant of BMDMs. (E) Relative mRNA levels of IL-1β in BMDMs. (F) TNF-α concentration in cell supernatant of BMDCs. (G) IL-6 concentration in cell supernatant of BMDCs. (H) Relative mRNA levels of IL-1β in BMDCs. (I) IL-10 concentration in cell supernatant of BMDMs. (J) IL-10 concentration in cell supernatant of BMDCs. ALPS: *A. muciniphila* LPS-treated cells group, ELPS: *E. coli* LPS-treated cells group. The cell experimental data shown was three replicates in one representative experiment. *ns* *p* > 0.05, **p* < 0.05, ***p* < 0.01, ****p* < 0.001. Unpaired-*t* test in A−J. *n* values: (A−J) n = 3.

**Figure S12.** *A. mucinniphila* LPS (ALPS) improved obesity and related glucose and lipid metabolism dysfunctions in DIO mice. (A) Body weight. (B) Plasma FFA. (C) Hepatic TG. (D) Hepatic TC. (E) Hepatic LDL. (F) Liver index. (G) Plasma insulin. (H) OGTT test. (I) AUC of OGTT. (J) AOC of OGTT. (K) ITT. (L) AUC of ITT. (M) AOC of ITT. (N) Plasma LPS. (O) Plasma TNF-α. (P) Plasma IL-6. (Q) Plasma IL-1β. (R) TNF-α/IL-10. (S) MCP-1. (T) IL-10. (U) Relative mRNA levels of ZO-1, ZO-2, Occludin, Claudin-1, and MUC-5 in ileum. MOD: Model group, high-fat diet (HFD). ALPS: *A. muciniphila* lipopolysaccharides, (ALPS)-treated HFD group. *ns* *p* > 0.05, **p* < 0.05, ***p* < 0.01, ****p* < 0.001. Welch’s *t*-test in D−J, O, and R; Unpaired *t*-test in A−C, K−N, P−Q, and S−T. *n* values: (A−T) *n* = 10. (U) *n* = 6.

**Figure S13.** The RNA-seq liver transcriptome analysis. (A) Volcano plots of differentially expressed genes in live. Each bubble represents a gene, and the color of the bubble represents a difference change (up-regulated, red; No significant change, black; down-regulated, green). (B) Heatmap of liver lipid metabolism-related gene expression in model group (MOD) and *A. mucinniphila* LPS (ALPS) group. (C) GO functional enrichment bubble map of down regulated gene sets by ALPS. (d) Relative mRNA levels of lipid metabolism-related genes in liver. MOD: Model group, high-fat diet (HFD). ALPS: *A. muciniphila* lipopolysaccharides, (ALPS)-treated HFD group. *ns* *p* > 0.05, **p* < 0.05, ***p* < 0.01, ****p* < 0.001. Unpaired *t*-test in C. *n* values: (D) *n* = 6, (A−C), *n* = 3.

**Figure S14.** Neutralization of IL-22 abrogated the hypoglycemic action of *A. mucinniphila* LPS (ALPS). (A) Hepatic TG. (B) Hepatic TG. (C) Hepatic LDL. (D) OGTT. (E) AUC of OGTT. (F) Plasma insulin. (G) ITT. (H) AUC of ITT. (I) HbA1C (%). *ns* *p* > 0.05, **p* < 0.05, ***p* < 0.01, ****p* < 0.001. One-Way ANOVA test followed by Dunnett's multiple comparisons tests in A−C, E and H; Kruskal-Wallis test followed by Dunn's multiple comparisons tests in C and F. *n* values: (A−I) *n* = 6.

**Figure S15.** *A. mucinniphila* LPS (ALPS) mainly functions through the TLR4 signaling pathway. (A) Western blotting of the TLR4, TLR2 and β-actin levels in BMDMs after incubating with ALPS or ELPS at the concentrations of 100 ng/mL for 24 h. (B) The ratio of the expression levels of TLR4/TLR2. ALPS: *A. muciniphila* LPS-treated cells group, ELPS: *E. coli* LPS-treated cells group. *ns* *p* > 0.05, **p* < 0.05, ***p* < 0.01. Unpaired *t*-test in B. *n* values: *n* = 3.

**Figure S16.** *A. mucinniphila* LPS (ALPS) promoted the production of short-chain fatty acids (SCFAs) in DIO mice. (A) Heatmap showed the production in DIO mice. (B) Fecal acetic acid (fold of MOD). (C) Fecal butyrate (fold of MOD). MOD: Model group, high-fat diet (HFD). ALPS: *A. muciniphila* lipopolysaccharides, (ALPS)-treated HFD group. *ns* *p* > 0.05, **p* < 0.05, ***p* < 0.01. Unpaired *t*-test in B. Mann–Whitney *U* test in C. *n* values: (B, C) *n* = 8.

**Figure S17.** *A. mucinniphila* LPS (ALPS)-altered gut microbiota protected high fat diet-induced metabolic disorders. (A) Body weight. F-HFD vs F-ALPS. (B) Plasma insulin. (C) HbA1C (%). (D) OGTT test. F-HFD vs F-ALPS. (E) AUC of OGTT. (F) AOC of OGTT. (G) ITT. F-HFD vs F-ALPS. (H) AUC of ITT. (I) AOC of ITT. (J) Hepatic TG. (K) TC. (L) LDL. (M) Plasma LPS. (N) Plasma TNF-α. (O) Plasma IL-6. (P) Plasma IL-1β. (Q) Plasma IL-10. (R) TNF-α/IL-10.(S)Colony forming units (CFUs) in feces cultured anaerobic in GAM medium. (T) CFUs in feces cultured aerobic in LB medium. F-HFD: The group of mice that received the feces of mice that were induced by a high-fat diet. F-ALPS: The group of mice that received the feces of mice that were treated by ALPS. F-HFD: The group of mice that received the feces of mice that were induced by a high-fat diet. F-ALPS: The group of mice that received the feces of mice that were treated by ALPS. *ns* *p* > 0.05, **p* < 0.05, ***p* < 0.01, ****p* < 0.001. Two-way ANOVA(Sidak) in A. Welch’s *t-*test in J, and P, Unpaired *t-*test in B−H, K−O, and Q−R; Mann–Whitney *U* test in I. *n* values: (A−T) *n* = 8.

**Figure S18.** Comparison of acute endotoxemia of ELPS and ALPS in C57BL/6J mice. (A) Experimental design showing groups and durations. (B) Body weight. (C) Percent survival (%). (D) Length of colon. (E) Representative images of gross appearance of the colon histology. (F) The representative H&E staining images of ileum pathological sections (scale bar, 50 μm). (G) Relative mRNA levels in ileum. (H) Plasma IL-6. (I) The relative mRNA levels of IL-6 in ileum. (J) Plasma IL-1β. (K) The relative mRNA levels of IL-1β in ileum. (L) Plasma TNF-α. (M) The relative mRNA levels of TNF-α in ileum. ELPS: *E. coli* LPS group, ELPS-treated C57BL/6J. ALPS: *A. muciniphila* LPS group, ALPS-treated C57BL/6J. *ns* *p* > 0.05, **p* < 0.05, ***p* < 0.01. One-Way ANOVA test followed by Dunnett's multiple comparisons tests in B, C, and J, K; Kruskal-Wallis test followed by Dunn’s multiple comparisons tests in L-M; Welch’s ANOVA test followed by Dunnett’s T3 multiple comparisons tests in I. *n* values: (B−D, G−M) *n* = 6.

**Figure S19.** The effects of ELPS and ALPS in DIO mice. (A) Experimental design showing groups and durations. (B) Body weight after 5 weeks. (C) Free diet blood glucose. (D) Plasma TC. (E) Plasma TG. (F) Plasma LDL. (G) Plasma IL-6. (H) Plasma IL-23. (I) Plasma IL-22. MOD: Model group, the high-fat diet group. ALPS: *A. muciniphila* LPS group, ALPS-treated high-fat diet group. ELPS: *E. coli* LPS group, ELPS-treated high-fat diet group. *ns* *p* > 0.05, **p* < 0.05, ***p* < 0.01, ****p* < 0.001. One-Way ANOVA test followed by Dunnett's multiple comparisons tests in B, and D−F, H-I; Kruskal-Wallis test followed by Dunn’s multiple comparisons tests in C, and G. *n* values: (B−I) *n* = 9.

**Figure S20.** ANI analysis between *A. muciniphila* HW07 and ATCC BBA-835.

**Figure S21.** Flow cytometry gating strategies. Gating strategies for IL-22^+^RORγt^+^ILC3 cells.

Table S1. Analysis of LPS biosynthetic genes in the genome of *Akkermansia muciniphila*.

| **Table S1. Analysis of LPS biosynthetic genes in the genome of *Akkermansia muciniphila*** | | | | | | | | | | | | | | | |
| --- | --- | --- | --- | --- | --- | --- | --- | --- | --- | --- | --- | --- | --- | --- | --- |
| Gene | Function | *E. coli* MG1655 | *A. muciniphila* HW07 | Identity (%) | Query cover (%) | E-value | *A. muciniphila* ATCC BAA-835 | Identity (%) | Query cover (%) | E-value | *A. muciniphila* HW07 | *A.muciniphila* ATCC BAA-835 | Identity  (%) | Query cover (%) | E-value |
| *lpxA* | acyl-ACP-UDP-N-acetylglucosamine O-acyltransferase | B1XD50 | JFJHEM_00188 | 48.3 | 87.02 | 4.5E-74 | B2ULY0 | 48.3 | 87.02 | 4.5E-74 | JFJHEM_00188 | B2ULY0 | 100 | 100 | 0 |
| *lpxB* | lipid-A-disaccharide synthase | B1XD51 | JFJHEM_02115 | 29.7 | 96.33 | 3E-48 | Q5LH14 | 29.7 | 96.33 | 3E-48 | JFJHEM_02115 | Q5LH14 | 100 | 100 | 0 |
| *lpxC* | UDP-3- O-acyl- N -acetylglucosamine deacetylase | B1XC73 | JFJHEM_01977 | 38.7 | 89.83 | 3.9E+54 | B2UNL5 | 38.7 | 89.83 | 3.9E+54 | JFJHEM_01977 | B2UNL5 | 100 | 100 | 0 |
| *lpxD* | UDP-3-O-(3-hydroxymyristoyl) glucosamine N-acyltransferase | P21645 | JFJHEM_00425 | 33.9 | 95.01 | 6.1E-51 | B2UND2 | 33.9 | 95.01 | 6.1E-51 | JFJHEM_00425 | B2UND2 | 100 | 100 | 0 |
| *lpxF* | lipid A 4'-phosphatase | P10441 | – | – | – | – | – | – | – | – | – | – | – | – | – |
| *lpxH* | UDP-2,3-diacylglucosamine diphosphatase | P43341 | – | – | – | – | – | – | – | – | – | – | – | – | – |
| *lpxK* | tetraacyldisaccharide 4'-kinase | B1X855 | JFJHEM_00598 | 30.1 | 96.64 | 9.3E-28 | B2UPD5 | 30.1 | 96.64 | 9.3E-28 | JFJHEM_00598 | B2UPD5 | 100 | 100 | 0 |
| *lpxL* | lauroyl/palmitoleoyl acyltransferase | P0ACV0 | JFJHEM_01585 | 23.7 |  | 0.00000012 | B2ULH5 | 23.7 |  | 0.00000012 | JFJHEM_01585 | B2ULH5 | 100 | 97 | 0 |
| *lpxM* | Lipid A biosynthesis myristoyltransferase | P24205 | – |  |  |  | – |  |  |  | – | – | – | – | – |
| *lpxP* | palmitoleoyl acyltransferase | P0ACV2 | JFJHEM_01585 | 21.9 | 86.93 | 0.00000012 | B2ULH5 | 21.9 | 86.93 | 0.00000012 | JFJHEM_01585 | B2ULH5 | 100 | 97 | 0 |

**Table S2.** Primers sequences in the experiments.

| Gene/protein name | Primer Sequence |
| --- | --- |
| *Gapdh* | Forward: 5'-AGGTCGGTGTGAACGGATTTG-3' |
|  | Reverse: 5'-TGTAGACCATGTAGTTGAGGTCA-3' |
| Occludin-1 | Forward: 5'-ATGTCCGGCCGATGCTCTC-3’ |
|  | Reverse: 5'-TTTGGCTGCTCTTGGGTCTGTAT-3’ |
| ZO-1 | Forward: 5'-ACCCGAAACTGATGCTGTGGATAG-3’ |
|  | Reverse: 5'-AAATGGCCGGGCAGAACTTGTGTA-3’ |
| ZO-2 | Forward: 5'-GGACACAATTCAGCATCAGCA-3' |
|  | Reverse: 5'-AAGTAGGACATGCGGTCTTCA-3' |
| MUC-5 | Forward: 5'-GTGGTTTGACACTGACTTCCC-3' |
|  | Reverse: 5'-CTCCTCTCGGTGACAGAGTCT-3' |
| Claudin-1 | Forward: 5'-GAAAGCCAGTAAATGTAGAGAGTTGA-3' |
|  | Reverse: ACTAAT-GTCGCCAGACCTGA-3' |
| *Tgfb1* | Forward: 5'-CAACCCAGGTCCTTCCTAAA-3' |
|  | Reverse: 5'-GGAGAGCCCTGGATACCAAC-3' |
| *Il17a* | Forward: 5'-TTTAACTCCCTTGGCGCAAAA-3' |
|  | Reverse: 5'-CTTTCCCTCCGCATTGACAC-3' |
| *Il22* | Forward: 5'-ATGAGTTTTTCCCTTATGGGGAC-3' |
|  | Reverse: 5'-GCTGGAAGTTGGACACCTCAA-3' |
| *Il23* | Forward: 5'-GGGGCTTTGGACACTGCTT-3' |
|  | Reverse: 5'-GTCTCGGCATCCTTGCATCTC-3' |
| *Il6* | Forward: 5'-CCGGAGAGGAGACTTCAC-3' |
|  | Reverse: 5'-TCCACGATTTCCCAGAGA-3' |
| *Il17f* | Forward: 5'-TGCTACTGTTGATGTTGGGAC-3' |
|  | Reverse: 5'-AATGCCCTGGTTTTGGTTGAA-3' |
| *Il2* | Forward: 5'-GTGCTCCTTGTCAACAGCG-3' |
|  | Reverse: 5'-GGGGAGTTTCAGGTTCCTGTA-3' |
| *Il19* | Forward: 5'-AGCCTGGATTGACAGGAA-3' |
|  | Reverse: 5'-GATAATCAGACGAGGCGTTTC-3' |
| *Il18* | Forward: 5'-GACTCTTGCGTCAACTTCAAGG-3' |
|  | Reverse: 5'-CAGGCTGTCTTTTGTCAACGA-3' |
| *Il10* | Forward: 5'-GCTCTTACTGACTGGCATGAG-3' |
|  | Reverse: 5'-CGCAGCTCTAGGAGCATGTG-3' |
| *Il1β* | Forward: 5'-TTGAAGAAGAGCCCATCCTC-3' |
|  | Reverse: 5'-CAGCTCATATGGGTCCGAC-3' |
| *Tnfα* | Forward: 5'-TAGCCAGGAGGGAGAACAGA-3' |
|  | Reverse: 5'-TTTTCTGGAGGGAGATGTGG-3' |
| *Ifng* | Forward: 5'-CACGGCACAGTCATTGAAAG-3' |
|  | Reverse: 5'-GCTGATGGCCTGATTGTCTT-3' |
| *Reg3g* | Forward: 5'-TTCCTGTCCTCCATGATCAAAA-3' |
|  | Reverse: 5'-CATCCACCTCTGTTGGGTTCA-3' |
| *Reg3b* | Forward: 5'-ATGCTGCTCTCCTGCCTGATG-3' |
|  | Reverse: 5'-CTAATGCGTGCGGAGGGTATATTC-3' |
| *S100a8* | Forward: 5'-TGTCCTCAGTTTGTGCAGAATATAAA-3' |
|  | Reverse: 5'-TCACCATCGCAAGGAACTCC-3' |
| *S100a9* | Forward: 5'-GGTGGAAGCACAGTTGGCA-3' |
|  | Reverse: 5'-GTGTCCAGGTCCTCCATGATG-3' |
| *Acot2* | Forward: 5'-GACAGGGTTTCTCTGTGTACC-3' |
|  | Reverse: 5'-GTGGCTTTACTCCCAGCACTT-3' |
| *Acot3* | Forward: 5'-CTGCTACATCCCTGGAGTTC-3' |
|  | Forward: 5'-CCCTTAACTGCTGAGCCATCTTT-3' |
| *Acot4* | Reverse: 5'-GCCTGTAACAGACATGGTAGATTC-3' |
|  | Reverse: 5'-CTGTAACAAGCACAGGCTGGTA-3' |
| *Cd36* | Forward: 5'-ATCTCAATGTCCGAGACTTTTCAAC-3' |
|  | Reverse: 5'-GCCAAGCTATTGGGACATGA-3' |
| *Acacb* | Forward: 5'-ACCCTAGTGCCGGCTCCTTCC-3' |
|  | Reverse: 5'-CCAGACATGCTGGGCCTCATAGT-3' |
| *Hmgcs1* | Forward: 5'-TGTTCTCTTACGGTTCTGGC-3' |
|  | Reverse: 5'-AAG TTCTCGAGTCAAGCCTTG-3' |
| *Cidea* | Forward: 5'-GCAGCCTGCAGGAACTTATC-3' |
|  | Reverse: 5'-TCATGAAATGCGTGTTGTCC-3' |
| *Elovl6* | Forward: 5'-AAAGCACCCGAACTAGGTGA-3' |
|  | Reverse: 5'-AGGAGCACAGTGATGTGGTG-3' |
| *Acox1* | Forward: 5'-CGCACATCTTGGATGGTAGT-3' |
|  | Reverse: 5'-GGC TTCGAGTGAGGAAGTTATAG-3' |
| *Lipe* | Forward: 5'-CATCAACCACTGTGAGGGTAAG-3' |
|  | Reverse: 5'-AAGGGAGGTGAGATGGTAACT-3' |
| *Pnpla2* | Forward: 5'-CTTTGTGGCACAGACCTCTAA-3' |
|  | Reverse: 5'-GGGAGTAGTCGATGGAGAAGATA-3' |
| **Primers for bacterium** | |
| *16S*  *(*reference gene*)* | Forward: 5'-ACTCCTACGGGAGGCAGCAGT-3' |
|  | Reverse: 5'-ATTACCGCGGCTGCTGGC-3' |
| *Frod* | Forward: 5'-CCGGGAATACGCTCTGGAAA-3' |
|  | Reverse: 5'-GCCAACCAACTAATGCACCG-3' |
| *SFB* | Forward: 5'-TGTGGGTTGTGAATAACAAT-3' |
|  | Reverse: 5'-GCGAGCTTCCCTCATTACAAGG-3' |
